# High‐Performance Ambipolar Organic Electrochemical Transistors Based on Diketopyrrolopyrrole‐Dialkoxybithiazole Conjugated Polymers for Single‐component Inverters

**DOI:** 10.1002/advs.202520003

**Published:** 2026-01-21

**Authors:** Jiazheng Li, Zhi Li, Jiayu Huang, Jingjing Su, Peijie Xu, Siwen Wang, Ping Zhang, Yuchuan Tian, Tao Pan, Junyang Liu, Junyu Li, Gang Ye, Ryan C. Chiechi, Yanxi Zhang, Wenjing Hong

**Affiliations:** ^1^ Institute of Flexible Electronics (IFE Future Technologies) State Key Laboratory of Physical Chemistry of Solid Surfaces & IKKEM College of Chemistry and Chemical Engineering Xiamen University Xiamen China; ^2^ Xiamen Key Laboratory of Stomatological Disease Diagnosis and Treatment Stomatological Hospital of Xiamen Medical College Xiamen China; ^3^ School of Electrical Engineering and Automation Jiangxi University of Science and Technology Ganzhou Jiangxi China; ^4^ Sinopec Shanghai Research Institute of Petrochemical Technology Shanghai China; ^5^ Hubei Key Laboratory of Polymer Materials Ministry of Education Key Laboratory for the Green Preparation and Application of Functional Materials School of Materials Science and Engineering Hubei University Wuhan China; ^6^ Department of Chemistry & Organic and Carbon Electronics Cluster North Carolina State University Raleigh North Carolina USA

**Keywords:** ambipolar organic electrochemical transistors, donor‐acceptor conjugated polymer, diketopyrrolopyrrole and dialkoxybithiazole, high‐spin state, single‐component inverter

## Abstract

Single‐component complementary inverters based on ambipolar organic mixed ionic‐electronic conductors (OMIECs) offer promise for simplifying logic circuit design and fabrication. However, the mismatched ambipolar transport properties in OMIECs have hindered the realization of such devices. To address this challenge, we designed two ambipolar OMIECs with high‐spin state: PDPPXO‐2TzC4 (where X = 3, 5). These donor‐acceptor (D‐A) conjugated polymers were synthesized through the copolymerization of diketopyrrolopyrrole (DPP) and dialkoxybithiazole (2Tz) units, incorporating modified ethylene glycol side chains. Both materials demonstrated outstanding and relatively balanced ambipolar OECT performance, achieving an on‐off ratio exceeding 10^5^ for both p/n‐type operations. PDPP3O‐2TzC4 achieved normalized maximum transconductances of 0.48 S cm^−1^ (p‐type) and 0.34 S cm^−1^ (n‐type), while PDPP5O‐2TzC4 reached 0.42 S cm^−1^ (p‐type) and 0.29 S cm^−1^ (n‐type). They also exhibited rapid and well‐matched p‐type and n‐type transient responses, with time constants (*τ*_on/off_) of less than 5 ms. Furthermore, leveraging the relatively balanced ambipolarity of PDPPXO‐2TzC4, we fabricated functional single‐component complementary inverters and obtained ternary logic characteristics under specific *V*_DD_. A biocompatibility assessment using human gingival fibroblasts (HGF) confirmed that PDPPXO‐2TzC4 is suitable for biological applications, laying a foundation for future OECT devices based on these materials in logic circuits and biomedical fields.

## Graphical abstract

Two donor‐acceptor conjugated polymers, incorporating diketopyrrolopyrrole (DPP) and dialkoxybithiazole (2Tz) units with ethylene glycol (EG) side chains, exhibit relatively balanced ambipolar organic electrochemical transistor (OECT) performance. This ambipolarity enables the realization of single‐component complementary inverters capable of ternary logic. Moreover, these polymers demonstrate good biocompatibility with human gingival fibroblasts (HGF), supporting their potential in bioelectronics.

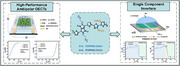

## 1 Introduction

Organic electrochemical transistors (OECTs) have significant potential for applications in flexible electronics, bioelectronics, and neuromorphic computing [1], owing to their high transconductance, effective ionic‐electronic coupling [2], and low power consumption [3]. The active channel materials in OECTs are organic mixed ionic‐electronic conductors (OMIECs), which effectively convert ionic signals into electrical signals [4]. Complementary inverters composed of p‐type and n‐type OECTs can be used to construct logic circuits [5], amplify physiological electrical signals [6], and create artificial neuron circuits [7, 8]. In recent years, n‐type OMIECs have gained significant attention and undergone rapid development, with their performance now approaching that of p‐type counterparts [9]. However, high‐performance balanced ambipolar OMIECs are still in short supply [10].

Ambipolar OMIECs, with their single‐component design, can reduce the complexity and cost of large‐scale OECT fabrication [11]. The primary challenge lies in the mismatched transport properties of the p‐type and n‐type operations. Donor‐acceptor conjugated polymers are designed to obtain an ideal frontier molecular orbital energy level for ambipolar OMIECs. Most reported ambipolar OMIECs are conjugated polymers incorporating strong electron‐withdrawing units, such as naphthalene diimide (NDI) and diketopyrrolopyrrole (DPP). While NDI‐based OMIECs typically exhibit superior n‐type performance over p‐type characteristics [12, 13], DPP‐based materials show asymmetric p‐ and n‐type behavior [14, 15]. In both cases, the ambipolar charge transport remains unbalanced, primarily due to suboptimal molecular orbital energy level alignment for efficient hole and electron conduction. Recently, a copolymer of bisistain‐lactone (BIL), a stronger electron‐withdrawing unit than NDI and DPP, and bithiophene [16], as well as a high‐spin thienoisoindigo‐based polymer [17], has been developed, both exhibiting balanced ambipolar behavior. Alternatively, blending p‐type and n‐type OMIECs has also been demonstrated as an effective strategy for achieving ambipolar OECTs [18]. We have developed a series of non‐fused, planar naphthalenediimide (NDI)‐dialkoxybithiazole (2Tz)‐based copolymers for ambipolar OECTs [19]. These materials exhibit excellent device performance [20] and remarkable operational stability [21], attributed to the deep lowest unoccupied molecular orbital (LUMO) level and high backbone planarity of the NDI‐2Tz system, making it a promising platform for OECT applications. However, their p‐type performance remains inferior to the n‐type characteristics. In order to achieve the ideal energy level for balanced ambipolar OECT materials, we propose replacing the too‐strong acceptor NDI unit with a moderately strong acceptor DPP unit to enhance p‐type performance and obtain balanced ambipolar OECT materials.

Here, we designed donor‐acceptor (D‐A) conjugated polymers by copolymerizing diketopyrrolopyrrole (DPP) and dialkoxybithiazole (2Tz). DPP was selected as the electron‐deficient moiety due to its high charge‐carrier mobility, which arises from its planar and rigid backbone and strong π–π stacking interactions [22]. To enhance ion transport and electrochemical doping, the DPP units were functionalized with triethylene glycol (TEG) and pentaethylene glycol (PEG) side chains. The polymers are named PDPP3O‐2TzC4 and PDPP5O‐2TzC4, respectively. The DPP‐dialkoxybithiophene‐based OECT exhibits predominantly p‐type behavior [23, 24]. Therefore, our strategy involves incorporating dialkoxybithiazole [25, 26], which is more electron‐deficient than dialkoxybithiophene (2Th), to enhance n‐type performance. The compact DPP and 2Tz result in a planar and rigid backbone structure. The electron‐deficient nature of both DPP and 2Tz moieties relative to the thiophene unit establishes this copolymer as a dual‐acceptor‐type conjugated polymer [27], where both building blocks contribute to n‐type charge transport characteristics.

As a result, PDPPXO‐2TzC4 (where X = 3, 5) exhibited exceptional and relatively balanced ambipolar OECT performance, achieving on‐off ratios (*I*_on/off_) exceeding 10^5^ for both p‐type and n‐type operations. The figure of merit, *µC* ^*^, was measured at 1.66/1.17 F cm^−1^ V^−1^ s^−1^ for PDPP3O‐2TzC4 and 1.27/1.07 F cm^−1^ V^−1^ s^−1^ for PDPP5O‐2TzC4 in p‐type and n‐type, respectively. PDPP3O‐2TzC4 achieved normalized maximum transconductances (*g*_m,norm_) of 0.48 S cm^−1^ (p‐type) and 0.34 S cm^−1^ (n‐type), while PDPP5O‐2TzC4 reached 0.42 S cm^−1^ (p‐type) and 0.29 S cm^−1^ (n‐type). The transient responses for both p‐type and n‐type are rapid and identical, with time constants (*τ*_on/off_) of less than 5 ms. Specifically, PDPP3O‐2TzC4 had p‐type time constants of *τ*_on/off_ = 3.75/0.85 ms, as well as n‐type values of *τ*_on/off_ = 3.81/1.01 ms. For PDPP5O‐2TzC4, the p‐type time constants were *τ*_on/off_ = 1.82/0.46 ms, while the n‐type values were *τ*_on/off_ = 1.85/0.85 ms. Furthermore, taking advantage of the balanced ambipolarity of PDPPXO‐2TzC4, we successfully fabricated single‐component complementary inverters based on both polymers and obtained ternary features. Ultimately, in vitro cytotoxicity testing using human gingival fibroblasts (HGF) confirmed that PDPPXO‐2TzC4 is biocompatible, establishing their potential use in logic circuits and bioelectronics.

## 2 Results

### 2.1 Characterization of polymers and OECTs

#### 2.1.1 The Material Design and Characterization

**FIGURE 1:**
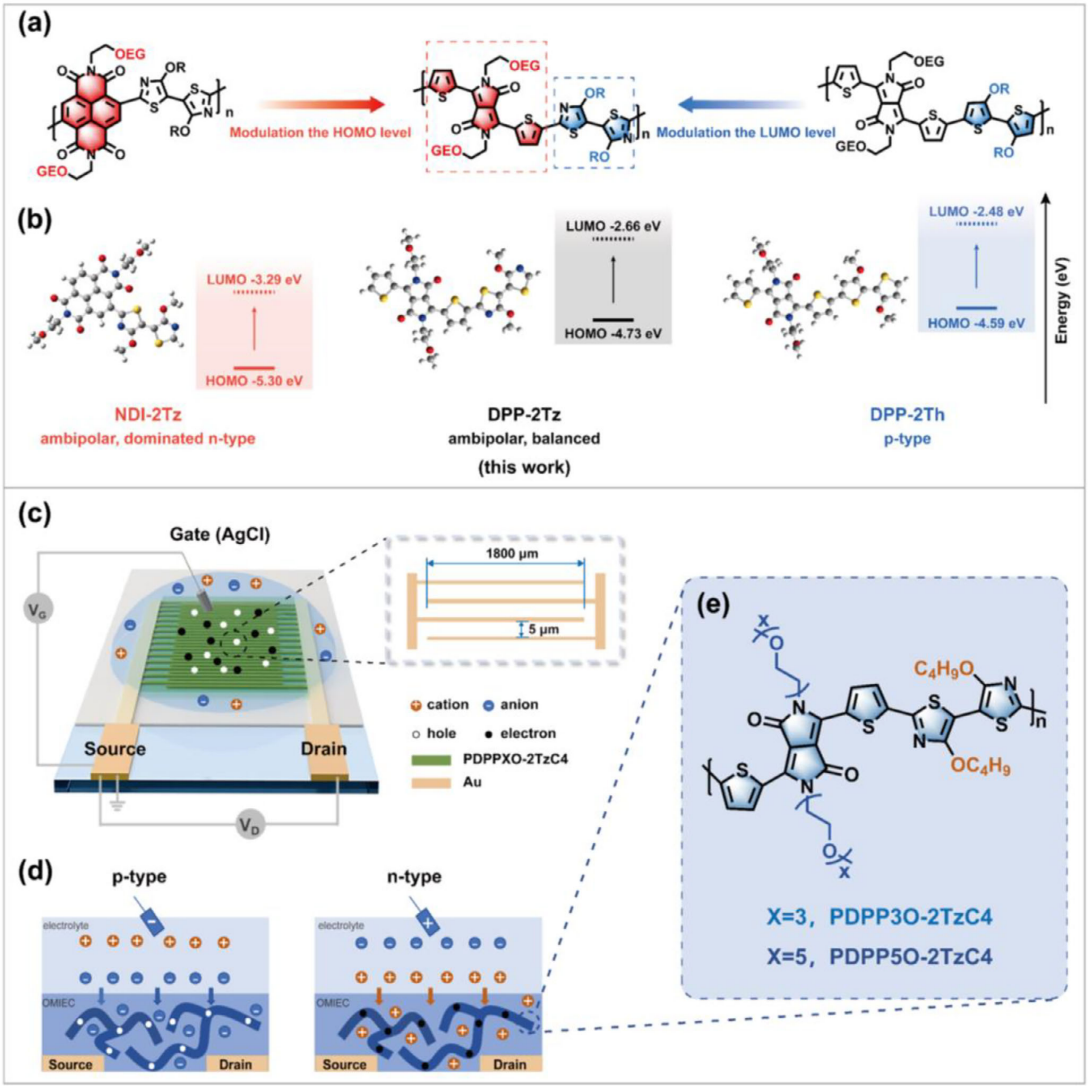
a) Rationale for polymer design. Chemical structures of NDI‐2Tz, DPP‐2Tz, and DPP‐2Th. b) Optimized molecular geometries and calculated energy levels of the NDI‐2Tz, DPP‐2Tz, and DPP‐2Th units. The DFT calculation was performed at the B3LYP/6‐31G(d,p) level. c) Schematic diagram of an OECT using interdigitated electrodes. d) Ion doping processes in p‐type and n‐type OECTs. e) Molecular structure of PDPP3O‐2TzC4 and PDPP5O‐2TzC4.

OMIECs were chosen as channel materials for OECTs because they can conduct both electrons and ions [4], which is crucial for optimal device performance. An appropriate bandgap is crucial for achieving ambipolarity, requiring both a low LUMO level (facilitating electron capture and transport for n‐type performance) and a high HOMO level (facilitating hole formation), which is the rationale of the polymers' design in this work (Figure 1a). Concurrently, DFT calculations in Figure 1b support that replacing the strong acceptor NDI with DPP and the strong donor 2Th with 2Tz results in ideal HOMO/LUMO energy levels and a narrow bandgap. Additionally, functioning as dual acceptors in comparison with thiophene, DPP, and 2Tz effectively lowers the LUMO energy level, reduces the bandgap, and enhances the potential for ambipolar behavior. OECTs operate by facilitating synergistic transport of ions and electrons, allowing transducing ionic signals into electronic currents. The structural diagram is shown in Figure 1c. These devices are categorized into p‐type and n‐type configurations based on their operational mechanisms (see Figure 1d). In n‐type OECTs, applying a positive gate voltage introduces cations from the electrolyte into the channel. These cations electrochemically dope the OMIECs and compensate for the electronic charges injected from the source electrode. This compensation introduces and stabilizes the charge carriers, thereby modulating the bulk conductivity of OMIECs and the drain current (*I*_D_). Conversely, p‐type OECTs operate with a negative gate voltage, which draws anions into the channel to compensate for holes, similarly tuning the *I*_D_ [28].

OMIECs for OECTs are predominantly p‐type materials, and the performance of these p‐type materials has been extensively investigated and is continuously advancing. In contrast, the library of high‐performance n‐type OMIECs remains limited, despite rapid progress in recent years. Most existing ambipolar systems are either blends of p‐type and n‐type materials that suffer from the performance degradation of their individual components, or they are single materials whose p‐type and n‐type operations are not sufficiently balanced for use in single‐component complementary circuits. PDPPXO‐2TzC4, the material used in this study (Figure 1e), exhibits excellent p‐type performance while also demonstrating notable n‐type behavior. Crucially, its p‐type and n‐type characteristics are well‐matched, making it an outstanding single‐component ambipolar material. The synthetic routes of the two polymers are shown in Figure S1.

In the polymer backbone, the DPP unit possesses a rigid planar bicyclic lactam structure, whose conjugated nature enhances the planarity of the polymer backbone. Meanwhile, planar backbone geometries of the two polymers were predicted using standard single‐determinant density functional theory (DFT) methods (Figure S2). This planar structure facilitates intramolecular charge transfer (ICT) and π–π stacking, thereby improving charge transport efficiency [29]. The dialkoxybithiazole structure within the polymer can synergize with the electron‐accepting properties of DPP, lowering the LUMO energy level of the polymer, reducing the bandgap, promoting electron injection and transport, while enhancing the material's ambipolar character. Introducing polar ethylene glycol (EG) side chains into the polymer endows the material with stronger hydrophilicity, facilitating the penetration of electrolyte ions (Na^+^, Cl^−^) into the polymer bulk. This balances the transport of charge carriers and ions, facilitating the charge compensation process [30]. The EG side chains endowed PDPPXO‐2TzC4 with excellent OECT performance. PDPP5O‐2TzC4, featuring longer EG side chains than PDPP3O‐2TzC4, exhibited superior performance in several aspects, such as lower threshold voltage (*V*_th_), improved stability, and faster response speed, etc. (Table 1). Simultaneously, the alkyl side chains (‐OC_4_H_9_) in the molecular backbone can reduce excessive swelling of the polymer, minimizing migration resistance for both charge carriers and ionic charges to enhance device performance [31].

**TABLE 1:** Aqueous electrolyte gated OECT characteristics of the polymers.

Polymer	Type	*I*_on/off_	*V*_th_ (V)	*g*_m, norm_ (S cm^−1^)	*τ*_on/off_ (ms)	*C* ^*^ (F cm^−3^)	µC* (F cm^−1^ V^−1^ s^−1^)	*µ* (×10^−3^ cm^2 ^V^−1^ s^−1^)
PDPP3O‐2TzC4	p‐type	> 10^5^	−0.57 ± 0.01	0.48 ± 0.01	3.75/0.85	134.01	1.66	12.39
	n‐type	> 10^5^	0.81 ± 0.02	0.34 ± 0.01	3.81/1.01	88.83	1.17	13.17
PDPP5O‐2TzC4	p‐type	> 10^6^	−0.43 ± 0.01	0.42 ± 0.01	1.82/0.46	166.67	1.27	7.62
	n‐type	> 10^6^	0.73 ± 0.01	0.29 ± 0.01	1.85/0.85	75.64	1.07	14.15

Cyclic voltammetry (CV) was performed (Figure S5), and the calculated LUMO/HOMO energy levels for PDPP3O‐2TzC4 were (−4.07/−5.21 V), and for PDPP5O‐2TzC4 were (−4.05 V/−5.23 V). Elevated HOMO energy levels in both PDPP3O‐2TzC4 and PDPP5O‐2TzC4 facilitate efficient hole carrier generation, consequently enabling enhanced p‐type operational characteristics in corresponding OECT devices. Both polymers exhibit narrow optical bandgaps of approximately 1.1 eV (Table 2), which facilitate donor‐acceptor interactions within the polymer backbones. This electronic structure further corroborates the ambipolar charge transport behavior observed in PDPP3O‐2TzC4 and PDPP5O‐2TzC4. In addition, CV measurements conducted in a 0.1 m NaCl aqueous electrolyte are presented in Figure S7.

**TABLE 2:** Optical properties, electrochemical properties, energy levels, and water contact angle (θ) of PDPP3O‐2TzC4 and PDPP5O‐2TzC4.

Polymer	λ_onset_, ^film^ [nm]	E_g_^opt.^ ^[a]^ [eV]	HOMO ^[b]^ [eV]	LUMO ^[c]^ [eV]	E_onset_^ox.^ ^[b]^ [V]	E_onset_^red.^ ^[c]^ [V]	Θ [°]
PDPP3O‐2TzC4	1126	1.10	−5.21	−4.07	0.61	−0.53	70.05 ± 1.15
PDPP5O‐2TzC4	1122	1.11	−5.23	−4.05	0.63	−0.55	66.55 ± 0.90

^[a]^ Optical bandgap calculated using the onset of the thin film absorption spectra (*E*_g_^opt^ = 1240/ λ_onset_).

^[b]^ Onset of CV oxidation recorded in a CHCN_3_ solution containing Bu_4_NPF_6_ electrolyte. HOMO =‐(5.10‐E_Fc/Fc_^+^+ E_onset_^ox.^) eV.

^[c]^ Onset of CV reduction recorded in a CHCN_3_ solution containing Bu_4_NPF_6_ electrolyte. LUMO =‐(5.10‐E_Fc/Fc_^+^+ E_onset_^red.^) eV.

To determine the number‐average molecular weight (M_n_) and polydispersity index (PDI) of PDPP3O‐2TzC4 and PDPP5O‐2TzC4, gel permeation chromatography (GPC) was performed using hexafluoroisopropanol (HFIP) as the eluent (Figure S8. The obtained M_n_ values are 12.7 kDa for PDPP3O‐2TzC4 and 11.7 kDa for PDPP5O‐2TzC4, respectively. The PDI is 3.38 for PDPP3O‐2TzC4 and 2.89 for PDPP5O‐2TzC4.

#### 2.1.2 The OECT Architecture

The OECTs devices tested in this experiment were based on interdigitated electrodes, as illustrated in Figure 1c. Compared to conventional electrodes, the interdigitated electrode design increases the width‐to‐length ratio by multiplying the width of each channel with the number of pairs, thereby substantially enhancing OECTs performance metrics, such as transconductance (*g*_m_). This relationship is described by the following Equation (1):

(1) $$g_{m}=\frac{\partial I_{D}}{\partial V_{G}}=\frac{Wd}{L}\times \mu \times C^{*}\times \left|\left(V_{th}-V_{G}\right)\right|$$

The channel width and length are represented by *W* and *L*, respectively; *d* is the thickness of the thin film, *µ* denotes the charge carrier mobility, *C* ^*^ is the volumetric capacitance, *V*_G_ stands for the gate voltage, and *V*_th_ is the threshold voltage. According to this equation, the interdigitated electrode design significantly increases *W* by leveraging multiple electrode pairs, thereby greatly enhancing *g*_m_. As a key metric of OECT performance, *g*_m_ reflects the device's ability to regulate the *I*_D_ via the gate voltage. High transconductance is a key hallmark of superior OECT performance, endowing the device with high sensitivity and strong signal amplification.

### 2.2 OECT Characterizations

**FIGURE 2:**
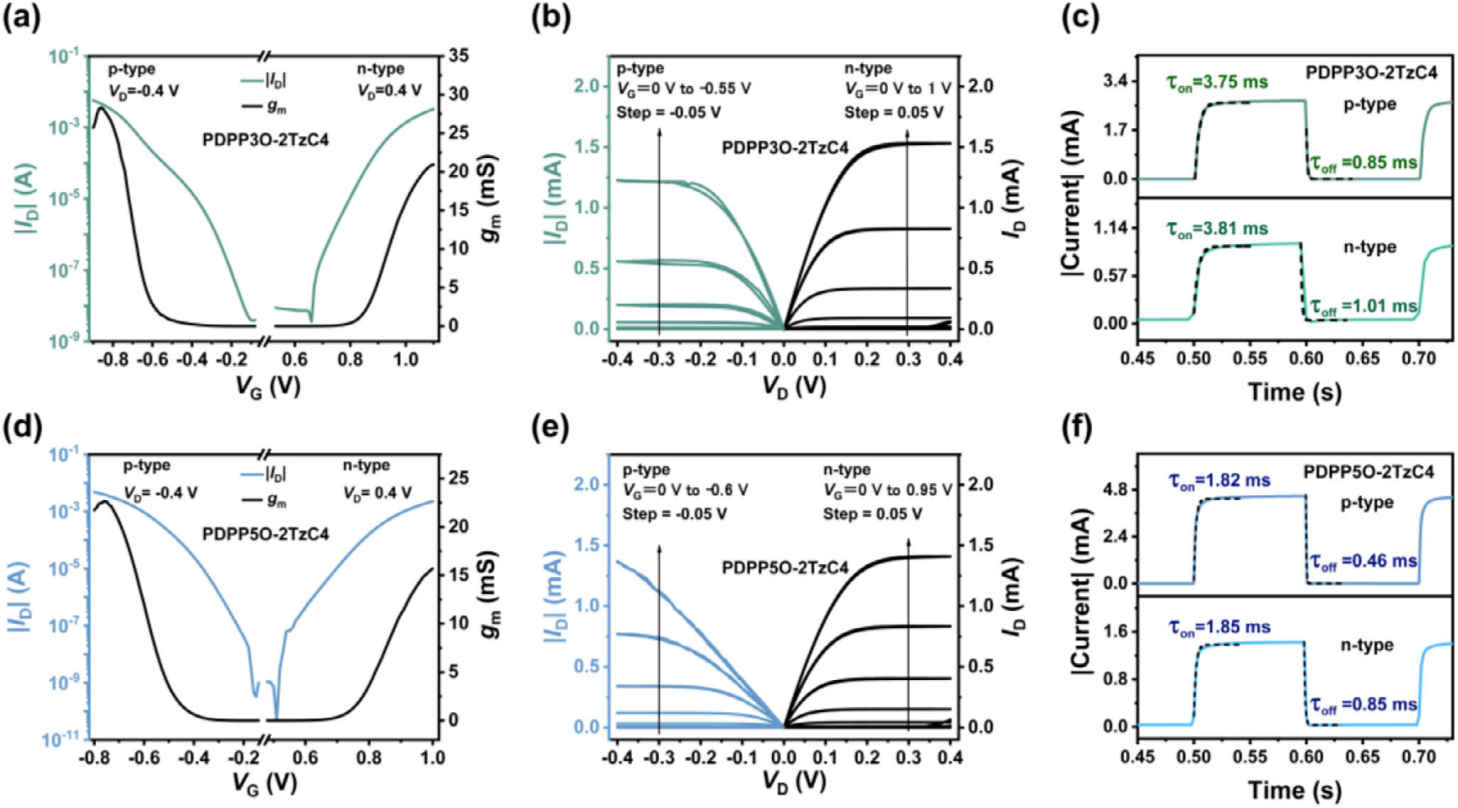
Ambipolar transfer curves of a) PDPP3O‐2TzC4 and d) PDPP5O‐2TzC4. Output curves of b) PDPP3O‐2TzC4 and e) PDPP5O‐2TzC4. Transient response of c) PDPP3O‐2TzC4 and f) PDPP5O‐2TzC4.

The performance of PDPPXO‐2TzC4 in OECTs was evaluated using interdigitated electrodes. The material solutions were spin‐coated onto the channel area at 1500 rpm, followed by annealing at 100°C for 30 min before testing. In an ambient air environment, we used 0.1 m NaCl as the electrolyte and Ag/AgCl as the gate electrode. The p‐type and n‐type transfer and output curves of PDPP3O‐2TzC4 and PDPP5O‐2TzC4 were measured by scanning the *V*_G_ at a rate of ±0.02 V/s, while maintaining a constant drain voltage (*V*_D_) of ±0.4 V. To ensure reliable experimental results, the transfer curves of multiple OECT devices were analyzed under the same conditions (Figure S9). Statistical analysis revealed negligible device‐to‐device variation in the transfer curves, indicating that the transistor characteristics are highly reproducible with minimal statistical deviation across the fabricated devices. As shown in Figure 2a,d, PDPP3O‐2TzC4 exhibits a high p/n‐type switching on‐off ratio exceeding 10^5^, whereas that of PDPP5O‐2TzC4 is even greater, surpassing 10^6^. The p‐type *V*_th_ of PDPP3O‐2TzC4 and PDPP5O‐2TzC4 is −0.57 and −0.43 V (Figure S10), respectively, which is associated with the electrochemical oxidation capabilities. A higher HOMO energy level corresponds to a lower ionization energy, facilitating electron ionization and thereby generating holes for p‐type conduction. The n‐type mode exhibits a higher *V*_th_ value than the p‐type, which is attributed to these polymers’ electrochemical reduction capabilities [32]. The n‐type *V*_th_ of PDPP3O‐2TzC4 and PDPP5O‐2TzC4 is 0.79 and 0.73 V, respectively. A higher LUMO makes the material more difficult to reduce, thereby hindering the device from reaching the on‐state at low gate voltages. The turn‐on voltages of the transistor in p‐type and n‐type modes are not fully symmetrical, indicating that the ambipolar performance is not perfectly balanced. There is still potential for improvement in ambipolarity through molecular engineering.

Subsequently, the *g*_m_ was obtained by differentiating the transfer curves according to Equation (1). The p‐type transconductance of PDPP3O‐2TzC4 reaches a maximum transconductance (*g*_m, max_) of 28.33 mS at *V*_G_ = −0.86 V, and the n‐type has a *g*_m_, _max_ of 20.91 mS (*V*_G_ = 1.1 V). The p/n‐type *g*_m, max_ of PDPP5O‐2TzC4 reached 22.68 and 15.70 mS at *V*_G_ of ‐0.76 and 1.0 V, respectively. To mitigate the structural influence on transconductance, *g*_m_ is normalized to obtain the normalized transconductance (*g*_m,norm_). The p/n‐type *g*_m,norm_ values for PDPP3O‐2TzC4 were 0.48/0.34 S cm^−1^, while for PDPP5O‐2TzC4 they were 0.42/0.29 S cm^−1^. Moreover, PDPP3O‐2TzC4 and PDPP5O‐2TzC4 showed excellent p‐type operational stability during transfer scans, with the *I*_D_ remaining nearly unchanged after 300 cycles, exhibiting minimal changes of only 3.04% and 10.18% for PDPP3O‐2TzC4 and PDPP5O‐2TzC4, respectively (Figure S13).

To probe the ionic‐electronic coupling capabilities, electrochemical impedance spectroscopy (EIS) was performed on both materials. The Bode plots (Figure S15) were fitted using an equivalent circuit model incorporating dual capacitive elements (Figure S17), enabling quantitative extraction of *C* ^*^. The p‐type and n‐type *C* ^*^ values were determined as 134.01/88.83 F cm^−3^ for PDPP3O‐2TzC4 and 166.67/75.64 F cm^−3^ for PDPP5O‐2TzC4. By applying Equation (1), the figure of merit *µC* ^*^ was calculated: 1.66/1.17 F cm^−1^ V^−1^ s^−1^ for PDPP3O‐2TzC4 and 1.27/1.07 F cm^−1^ V^−1^ s^−1^ for PDPP5O‐2TzC4, corresponding to p‐type and n‐type operation, respectively. Charge carrier mobilities (*µ*) were subsequently derived from these parameters, as shown in Table 1.

The transient responses of the OECTs were assessed by applying gate voltage pulses while maintaining a constant *V*_D_, as shown in Figure 2c,f. For the n‐type OECT, a gate voltage of 1 V was applied, while for the p‐type, a gate voltage of ‐0.8 V was used. The measured turn‐on/turn‐off time constants (*τ*_on/off_) for PDPP3O‐2TzC4 were 3.75 and 0.85 ms, respectively, for the p‐type, and 3.81 and 1.01 ms for the n‐type. In the case of PDPP5O‐2TzC4, the p‐type *τ*_on/off_ were found to be 1.82 and 0.46 ms, while the n‐type values were 1.85 and 0.85 ms. Both materials demonstrated fast ambipolar transient responses. This rapid switching capability allows OECTs to process high‐speed signals. Consequently, they are highly advantageous for applications that require quick response times.

Both materials showcased good p‐type operational stability (see Figure S18). Notably, PDPP3O‐2TzC4 and PDPP5O‐2TzC4 retained 93% of their drain current after 100 p‐type pulsing cycles. Extended cycling tests revealed that PDPP5O‐2TzC4 displayed superior stability, with only a one‐order‐of‐magnitude current reduction in current after 1500 cycles. In contrast, PDPP3O‐2TzC4 experienced an about two‐order‐of‐magnitude decrease after 1500 cycles. This difference in degradation behavior indicates that the longer ethylene glycol side chains significantly enhance operational stability. Due to their high LUMO energy levels and n‐type threshold voltages, the n‐type stability of both materials was less ideal, with the *I*_D_ of PDPP3O‐2TzC4 and PDPP5O‐2TzC4 decreasing by 73% and 56%, respectively, after 100 pulsing cycles (Figure S19).

### 2.3 Polymer Thin‐Film Characterizations

**FIGURE 3:**
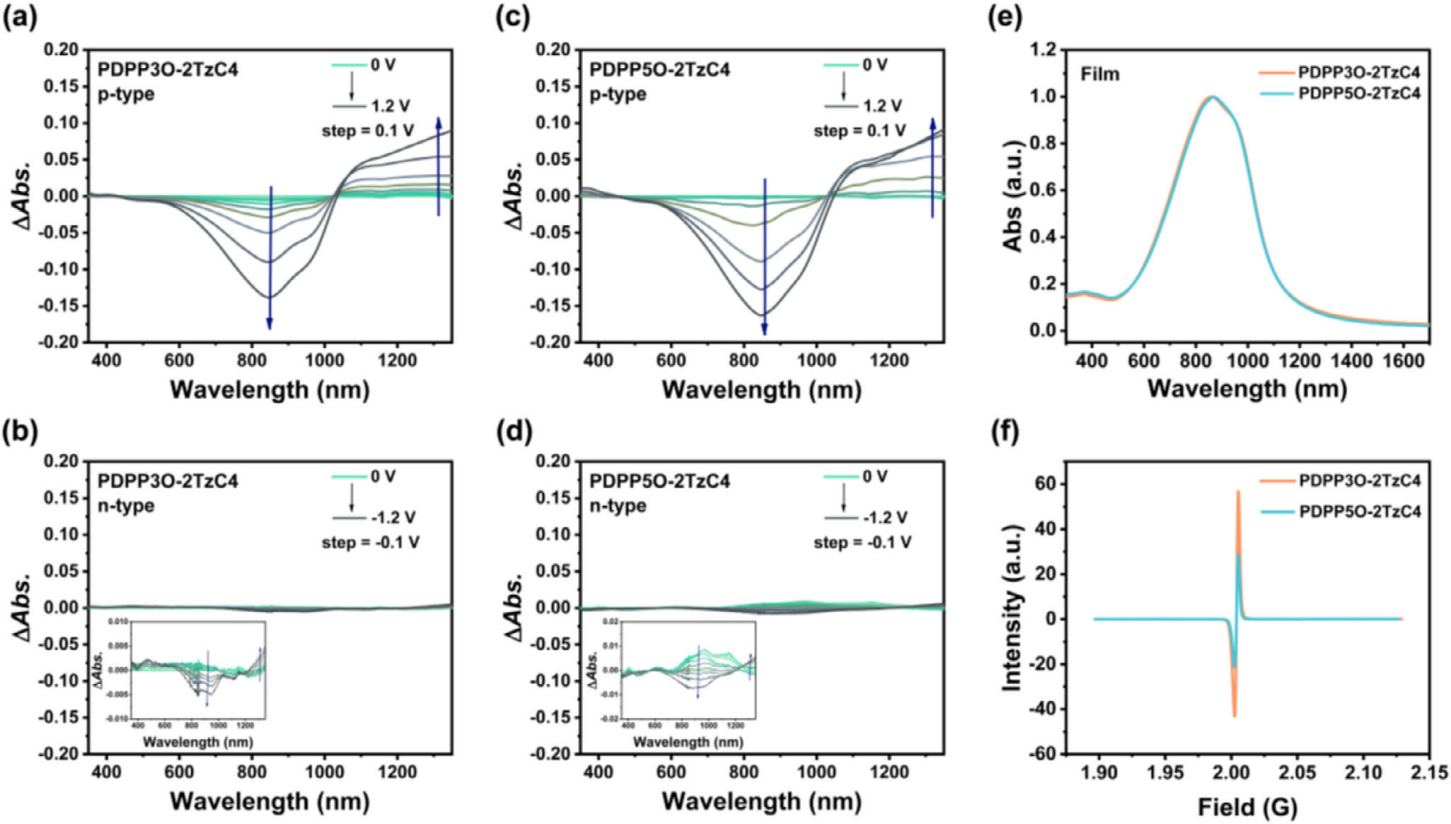
a–d) Electrochemical spectra of p‐type and n‐type of PDPP3O‐2TzC4, PDPP5O‐2TzC4. The Δ*Abs*. indicates the difference in absorption intensity from 0 V at different bias voltages. e) Normalized UV‐vis‐NIR absorption spectra of PDPP3O‐2TzC4 and PDPP5O‐2TzC4 films. f) Room temperature EPR signals of two polymers in a solid.

To investigate the electrochemical doping states (in 0.1 m NaCl electrolyte) and optoelectronic properties of PDPP3O‐2TzC4 and PDPP5O‐2TzC4, we conducted electrochemical spectroscopy. Both polymers display dual‐band absorption characteristics due to their D‐A structure, featuring ICT absorption in the range of 600 to 1000 nm and π–π^*^ transitions between 300 and 500 nm. Figure 3a,c shows the absorption changes that occur when oxidation potentials are applied to both polymers. Significant changes were observed only after the bias reached 0.7 V. When the oxidation potential was increased to 0.8 V, a pronounced attenuation of the ICT band between 600–1000 nm was observed (manifested as negative Δ*Abs*. values). The p‐type doping results in the presence of hole polarons, as evidenced by the intensified polaron absorption near 1200 nm. As the oxidation potential was further raised to 1.2 V, the neutral absorption of this polymer was almost completely bleached out, indicating that the film had been oxidized to a highly p‐type doped state [32]. The absorption changes were less pronounced when reduction potentials were applied to both polymers, as shown in Figure 3b,d. Both PDPP3O‐2TzC4 and PDPP5O‐2TzC4 exhibited minor changes in ICT absorption upon the application of reduction potentials (0 to ‐1.2 V). The optical bandgaps of PDPP3O‐2TzC4 and PDPP5O‐2TzC4, calculated from the onset of their thin‐film UV‐vis‐NIR (Ultraviolet‐visible‐Near Infrared) absorption spectra (Figure 3e), are 1.10 and 1.11 eV, respectively. A maximum absorption peak at 869 nm was observed in thin films for both PDPP3O‐2TzC4 and PDPP5O‐2TzC4. Their solution‐phase absorption spectra are shown in Figure S21.

Electron paramagnetic resonance (EPR) spectroscopy was conducted on PDPP3O‐2TzC4 and PDPP5O‐2TzC4 at room temperature (Figure 3f). The room temperature EPR signal indicates the presence of unpaired electrons in the material, reflecting high‐spin state characteristics, which are typically associated with an open‐shell electronic structure. This is beneficial for achieving balanced ambipolar transport [17]. CASSCF calculations suggest that the PDDP‐2Tz conjugated polymer exhibits a radicaloid ground state (Figure S4). These calculations indicate that the polymer possesses unpaired electrons, and EPR measurements demonstrate that it has non‐zero spin states. Therefore, there is a significant possibility that these polymers are open‐shell.

**FIGURE 4:**
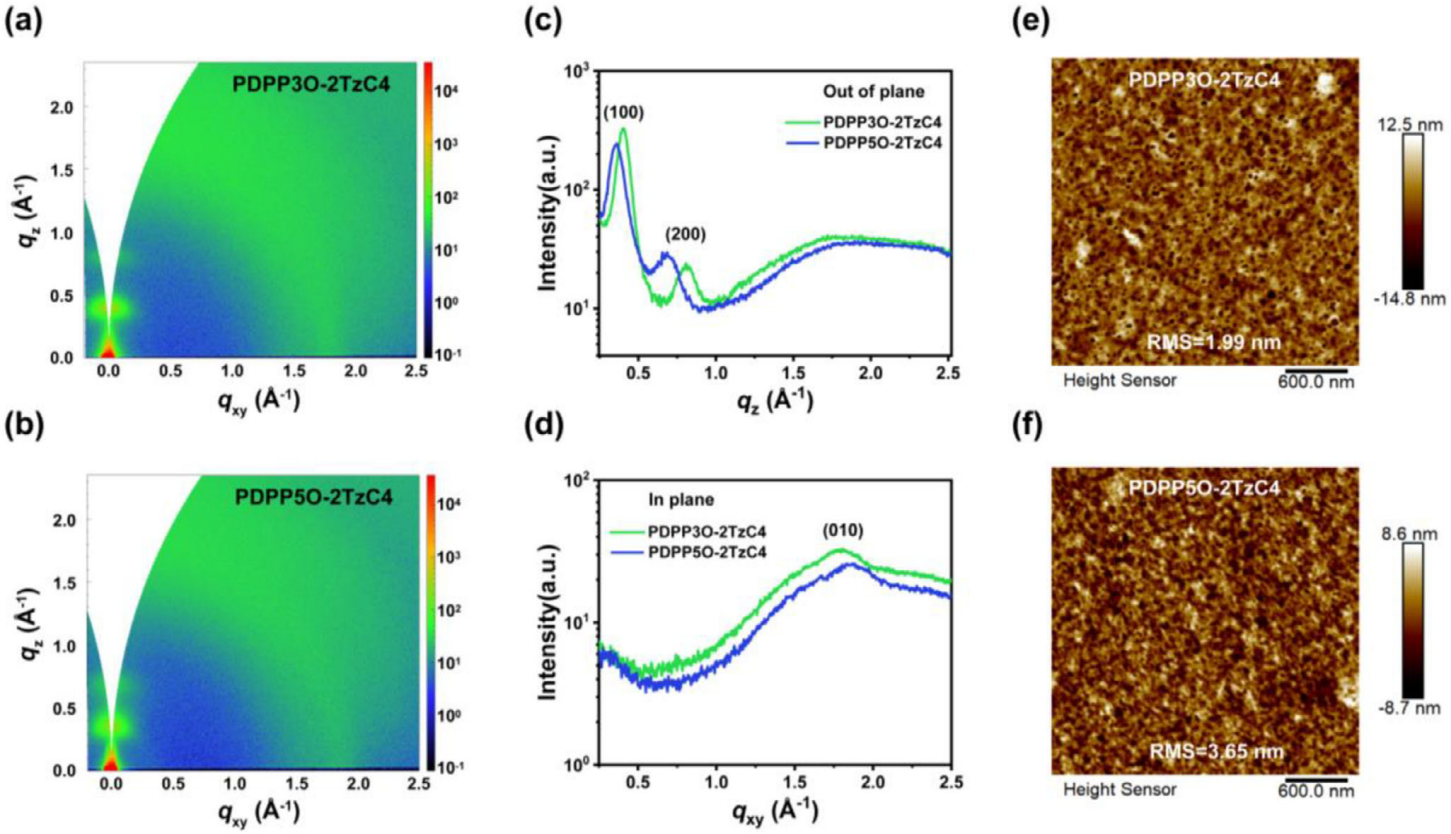
2D GIWAXS patterns of a) PDPP3O‐2TzC4 and b) PDPP5O‐2TzC4. Line‐cut profiles of PDPP3O‐2TzC4 and PDPP5O‐2TzC4 thin films: c) integration along the out‐of‐plane (*q*_z_) and d) in‐plane (*q*_xy_) directions. AFM height images of e) PDPP3O‐2TzC4 and f) PDPP5O‐2TzC4 films.

The stacking structure of the polymer thin films was analyzed using Grazing‐Incidence Wide‐Angle X‐ray Diffraction (GIWAXS). According to Figure 4a–d, PDPP3O‐2TzC4 and PDPP5O‐2TzC4 exhibit similar orientations and both demonstrate good crystallinity. These two polymers primarily adopt an edge‐on orientation. The PDPP3O‐2TzC4 and PDPP5O‐2TzC4 films show distinct lamellar (100) diffraction peaks at *q*_z_ = 0.40 Å^−1^ (d‐spacing = 15.71 Å) and *q*_z_ = 0.36 Å^−1^ (d‐spacing = 17.45 Å) in the out‐of‐plane (OOP) direction, respectively. This indicates that the longer EG chains increase the lamellar spacing. These expanded spacing may facilitate more efficient ion transport, thereby contributing to the faster response speed of PDPP5O‐2TzC4 [30]. Additionally, the calculated π–π stacking distances are 3.47 Å for PDPP3O‐2TzC4 and 3.38 Å for PDPP5O‐2TzC4, showing only a minimal variation. This minor difference demonstrates that the length of side chains has a smaller effect on the π–π stacking distance compared to its influence on lamellar spacing. The morphologies of both polymer films were further examined using Atomic Force Microscopy (AFM). These two polymers exhibited porous surface structures. These structures might facilitate ion injection while maintaining electron transport, thereby enhancing OECT performance. The root mean square (RMS) roughness values were 1.99 nm for PDPP3O‐2TzC4 and 3.65 nm for PDPP5O‐2TzC4 (Figure 4e,f).

### 2.4 Single‐Component Inverters

**FIGURE 5:**
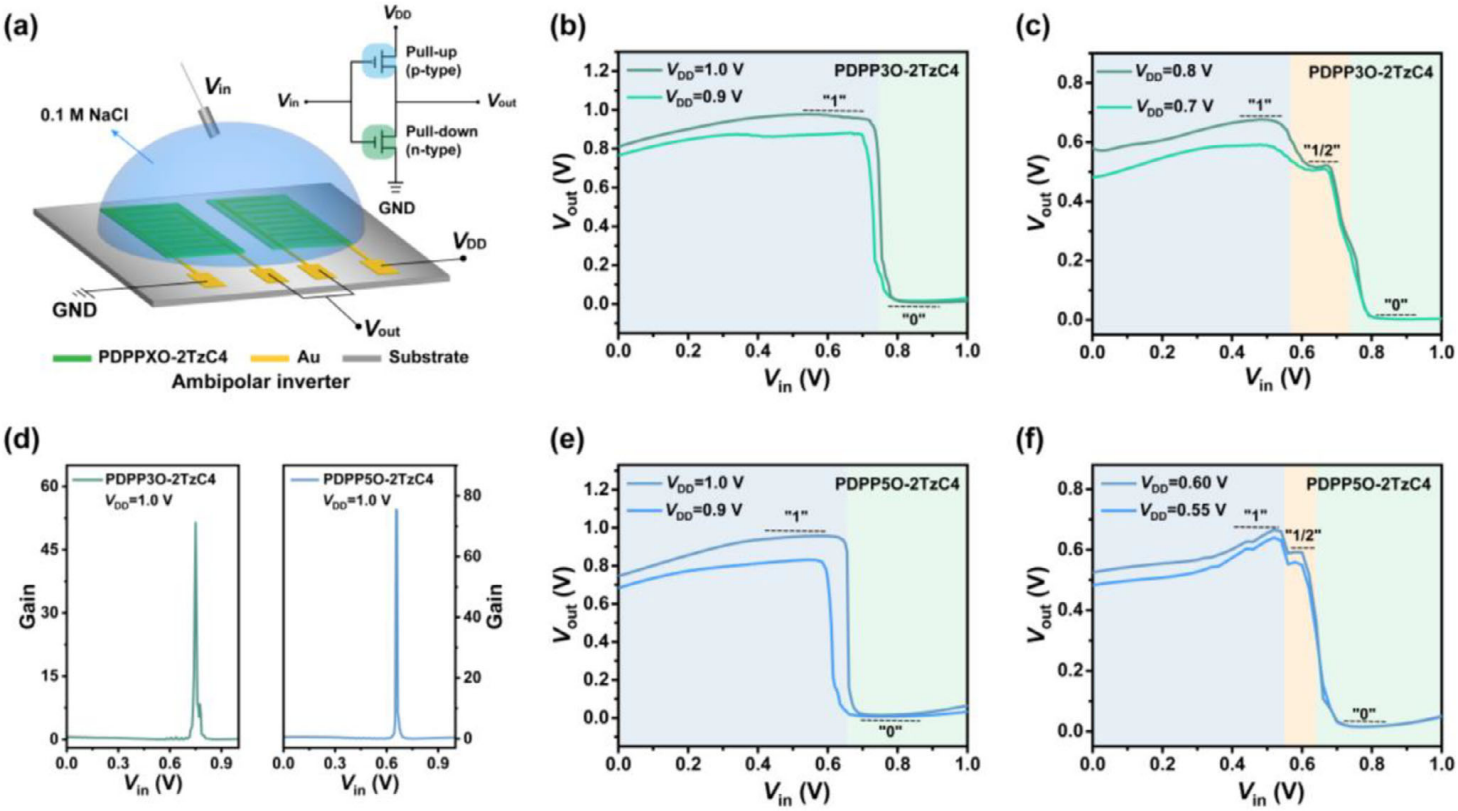
Ambipolar inverters based on PDPP3O‐2TzC4 and PDPP5O‐2TzC4. a) Structure and corresponding circuit schematic of the ambipolar inverter. b, c) Voltage transfer curves of a PDPP3O‐2TzC4‐based inverter at *V*_DD_ = 1.0 and 0.9 V, 0.8 and 0.7 V respectively. d) Voltage gain characteristics of single‐component inverters based on PDPP3O‐2TzC4 and PDPP5O‐2TzC4 at *V*_DD_ = 1.0 V. e, f) Voltage transfer curves of the PDPP5O‐2TzC4‐based inverter at *V*_DD_ = 1.0 and 0.9 V, 0.60 and 0.55 V respectively.

Ambipolar materials enable the fabrication of single‐component inverters, allowing the complementary logic circuit to be patterned using just one material (see Figure 5a). This approach eliminates the need for dual‐material deposition common in traditional p‐n complementary circuits, thereby significantly reducing production costs and complexity. Inverters serve various functions, such as amplifying weak electrical signals like electrophysiological signals and enhancing the sensing sensitivity (e.g., biosensors). To assess the feasibility of using PDPP3O‐2TzC4 and PDPP5O‐2TzC4 in single‐component inverters, devices were fabricated and tested. The results demonstrated a good p‐type and n‐type matching of both materials, confirming that functional single‐component inverters based on PDPP3O‐2TzC4 and PDPP5O‐2TzC4 could be successfully realized, as illustrated in Figure 5b–f. Figure 5b,e presents the voltage transfer characteristics (VTC) of PDPP3O‐2TzC4 and PDPP5O‐2TzC4‐based inverters at *V*_DD_ = 0.9 and 1.0 V, respectively. Both polymers exhibited well inverter characteristics, achieving substantial gains of 51.37 V/V and 75.40 V/V at *V*_DD_ = 1.0 V, as quantified in Figure 5d. Furthermore, single‐component inverters based on these two polymers demonstrated ternary logic operations at a specific *V*_DD_. The PDPP3O‐2TzC4‐based inverter notably exhibited three distinct plateaus at *V*_DD_ = 0.7 and 0.8 V (Figure 5c). Each plateau can be assigned a logic designation of “1”, “1/2”, and “0”, which allows for the implementation of ternary logic. This enables a single device to process three distinct states of information, surpassing the capabilities of binary systems. As a result, it significantly enhances information density and reduces circuit complexity [33, 34]. The appearance of a plateau in the intermediate region of the VTC curve can be attributed to the fact that, when the effective input voltage (*V*_in_) is between the threshold voltages of the p‐type and n‐type channels, both channels are either off or only slightly on. In this scenario, the output voltage (*V*_out_) is determined by the voltage division between the resistances of the two channels. This leads to an intermediate output voltage that is neither a logical high nor a logical low, resulting in a “1/2” state. Specifically, when the *V*_in_ lies between the p‑type and n‑type threshold voltages, approximately −0.57 and 0.79 V for PDPP3O‑2TzC4, the effective gate voltage applied to the channel is insufficient to induce significant electrochemical oxidation or reduction of the polymer. As a result, the material remains in a non‐doped or partially doped state. The resistances of the two channels, which are based on ambipolar materials, are comparable, leading to a division of the output voltage. The same principle applies to the intermediate plateau observed in inverters based on PDPP5O‑2TzC4. As *V*_in_ further increases, the n‐type OECT experiences increasing charge carrier concentration and enters its conduction regime, ultimately driving the *V*_out_ to low‐level logic states. Meanwhile, as shown in Figure 5f, the VTC curves of PDPP5O‐2TzC4‐based inverters similarly displayed three distinct plateaus, demonstrating ternary logic characteristics. Finally, the hysteresis of the VTC curves for both polymers is presented in Figure S24.

### 2.5 In Vitro Cytotoxicity Testing

**FIGURE 6:**
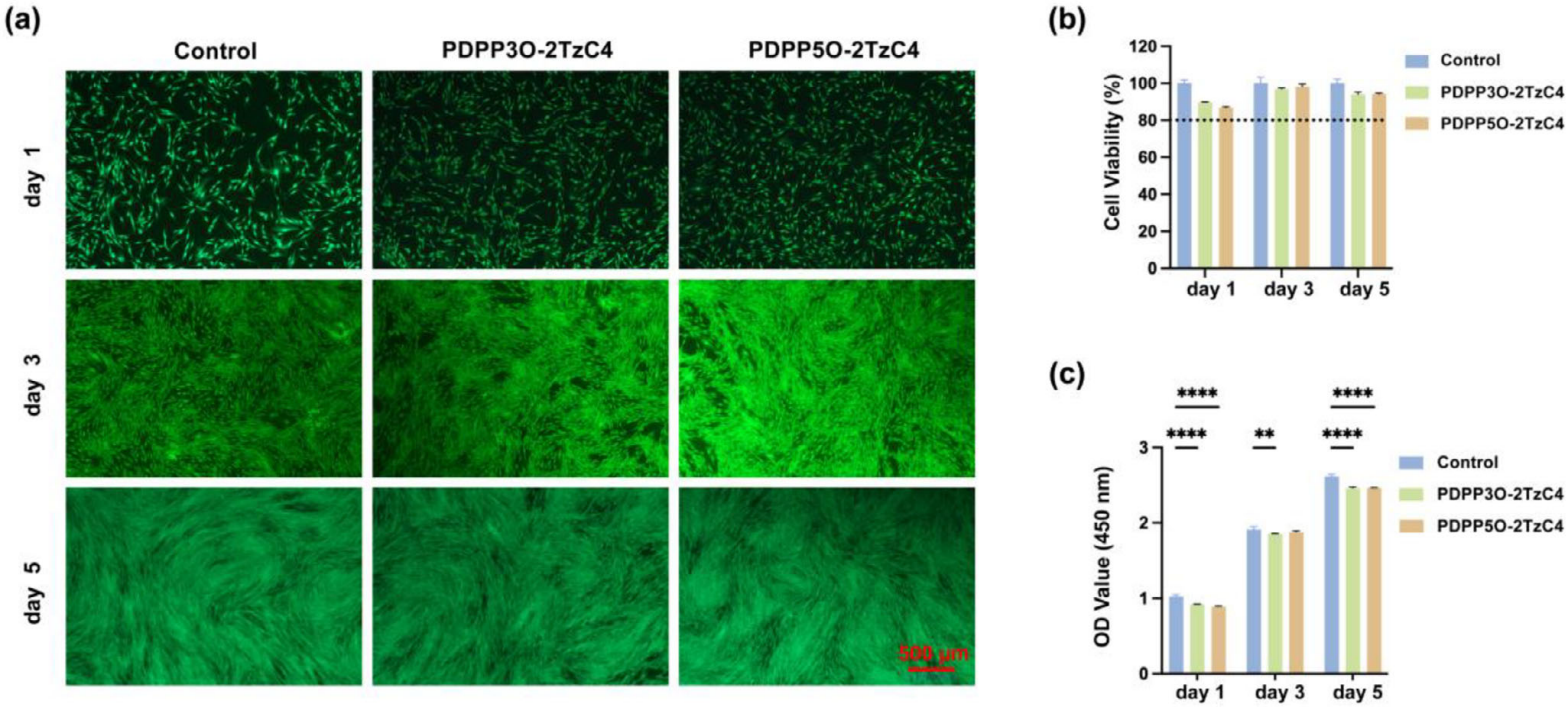
The biocompatibility of PDPP3O‐2TzC4 and PDPP5O‐2TzC4 was evaluated using live/dead staining. a) Fluorescent images of HGF live cells stained green by Calcein AM and dead cells stained red by propidium iodide (PI). Quantitative analysis of b) cell viability and c) cell proliferation co‐cultured with different specimens (n = 3 independent replicates, mean ±SD). ^**^ *p* value < 0.01, and ^****^ *p* value < 0.0001.

Human gingival fibroblasts (HGF) were directly seeded onto the cell culture plate (control group) and glass slides coated with PDPP3O‐2TzC4 or PDPP5O‐2TzC4 thin films, and cultured for 1, 3, and 5 days, respectively. The effects of the two polymers on cells were investigated using live/dead staining, where green fluorescence indicates live cells, red fluorescence indicates dead cells. As shown in Figure 6a, the cell viability and growth trends observed at 1, 3, and 5 days were similar to those of the control group, with mostly green fluorescence and almost no red fluorescence. This suggests normal cell growth morphology and demonstrates that both polymers had no significant adverse impact on cell proliferation or viability [35]. As illustrated in Figure 6b,c, throughout the experimental period (days 1, 3, and 5), the cell viability in both material groups exceeded 80% and was not significantly lower than that of the control group. As reported by ISO 10993–5 (ISO, 2009), percentages of cell viability above 80% are considered non‐cytotoxic. The proliferation rates were similar to those of the control group. The optical density (OD) values increased synchronously over time, indicating that the cells successfully adhered to the material surfaces and maintained stable viability and metabolism. The OD value on day 1 confirmed initial cell attachment, while the steady increase in OD values during subsequent culture indicated that the cells maintained a healthy physiological state. This confirms that the materials did not interfere with the normal cell proliferation cycle (based on three independent replicate experiments) [36]. According to conventional safety evaluation criteria for biomaterials, PDPP3O‐2TzC4 and PDPP5O‐2TzC4 exhibit no biological toxicity and meet biocompatibility requirements. Electronic devices based on these two polymers show promise for applications in biomedical fields such as biosensing.

## 3 Conclusion

We developed two high‐spin polymers, PDPP3O‐2TzC4 and PDPP5O‐2TzC4, based on the DPP and 2Tz D‐A polymer backbone and featuring EG and alkyl side chains, for use in ambipolar OECTs. Their structures, properties, electrochemical doping states, and performance in OECT devices were thoroughly investigated. The D‐A backbone engineering, incorporating DPP and 2Tz moieties, not only narrows the bandgap but also enhances the planarity of the polymer backbone. Concurrently, side‐chain engineering improves the charge carrier injection efficiency and balances the cation and anion coupling to the polymer backbone. This synergistic effect results in an n‐type performance that matches the leading p‐type properties, enables balanced ion doping, and facilitates both electron and hole transport within a single‐component material. Therefore, OECTs based on PDPPXO‐2TzC4 demonstrate outstanding performance, a significant achievement given the scarcity of high‐performance ambipolar materials. The on‐off ratio exceeded 10^5^ for both p‐ and n‐type operations. The normalized maximum transconductance for p/n‐type reached 0.48/0.34 S cm^−1^ for PDPP3O‐2TzC4 and 0.42/0.29 S cm^−1^ for PDPP5O‐2TzC4, respectively, along with short transient response times. Furthermore, single‐component inverters were successfully constructed using PDPP3O‐2TzC4 and PDPP5O‐2TzC4, and both exhibited ternary logic behavior under specific *V*_DD_, paving the way for simplified logic circuits and higher information density. Finally, biocompatibility tests confirmed the non‐toxicity of both materials in human cell experiments, laying a foundation for their biomedical applications. These advances represent a significant step toward large‐scale, single‐component inverters. A key future goal is to enhance the n‐type performance to match the p‐type capabilities in all aspects, such as by lowering threshold voltages and improving operational stability. The realization of this goal necessitates a systematic approach, encompassing both the optimization of material parameters (including molecular weight, EG chain length/ratio) and device architecture, with the ultimate aim of achieving high‐gain, high‐stability, multi‐value, single‐component inverters.

## 4 Experimental Section

### 4.1 Materials

The synthetic details of PDPPXO‐2TzC4 (where X = 3, 5) are described in Supporting Information. All reagents and solvents were commercial and were used as received. 4,4'‐dibutoxy‐2,2'‐bis(trimethylstannyl)‐5,5'‐bithiazole (**2Tz‐C4**), 3,6‐bis(5‐bromothiophen‐2‐yl)‐2,5‐bis(2‐(2‐(2‐methoxyethoxy)ethoxy)ethyl)‐2,5‐dihydropyrrolo[3,4‐c]pyrrole‐1,4‐dione (**DPP‐3O‐br**) and 3,6‐bis(5‐bromothiophen‐2‐yl)‐2,5‐di(2,5,8,11,14‐pentaoxahexadecan‐16‐yl)‐2,5‐dihydropyrrolo[3,4‐c]pyrrole‐1,4‐dione (**DPP‐5O‐br**) were purchased from Suna Tech. Inc. 1,1,1,3,3,3‐Hexafluoro‐2‐propanol (HFIP), etonitrile were purchased from Shanghai Acmec Biochemical Technology Co., Ltd.

### 4.2 Interdigitated Electrode Fabrication

To characterize the charge transport properties of PDPP3O‐2TzC4 and PDPP5O‐2TzC4, interdigitated electrodes (source and drain electrodes arranged in an interpenetrating comb structure) were fabricated. The interdigitated electrode pattern was defined using standard photolithography. Specifically, the first layer of the electrode pattern was created on a glass substrate by photolithography using AE5214E photoresist and a wafer‐scale mask aligner, followed by development. Subsequently, a 50 nm thick Au layer (with a 3 nm Cr adhesion layer) was deposited onto the patterned AZ5214E photoresist via thermal evaporation to form the second layer. Finally, an SU‐8 photoresist layer was patterned as the third, insulating layer, selectively exposing only the electrode channel regions. The resulting devices featured a channel width (*W*) of 1800 µm, a channel length (*L*) of 5 µm (Figure S27), and comprised 15 pairs of source/drain electrodes.

### 4.3 OECT Fabrication and Characterization

Prior to testing, the electrodes were ultrasonically cleaned in ethanol for 1–3 min, dried under a nitrogen stream, and subsequently treated with UV‐ozone for 30 min. Then, an 8 µL volume of the polymer solution PDPPXO‐2TzC4 dissolved in hexafluoroisopropanol, concentration: 5 mg/mL) was spin‐coated onto the channel region at 1500 rpm for 30 s, followed by annealing on a hot plate at 100°C for 30 min. During device characterization, an Ag/AgCl electrode served as the gate electrode (top gate), immersed in a 0.1 m NaCl aqueous electrolyte solution. For p‐type devices, the drain voltage (*V*_D_) was fixed at ‐0.4 V, while for n‐type devices, *V*_D_ was set to 0.4 V. The gate voltage (*V*_G_) was swept from 0 V to the voltage corresponding to the maximum transconductance. All measurements were performed in ambient using a Keithley 2612B SourceMeter.

### 4.4 In Vitro Cytotoxicity Testing

Under the same conditions used for fabricating OECT devices, PDPP3O‐2TzC4 and PDPP5O‐2TzC4 were spin‐coated onto glass slides to prepare thin‐film samples. The samples were then subjected to rigorous sterilization procedures, which involved immersion in alcohol for 30 min, followed by UV irradiation on both sides for 1 h each, and a final sterilization with ethylene oxide. The sterilized blank glass slides, as well as those coated with PDPP3O‐2TzC4 and PDPP5O‐2TzC4 thin films, were placed in a 24‐well cell culture plate. Human gingival fibroblasts (HGF) were seeded onto the surface of each sample. The cells were cultured for 1, 3, and 5 days before testing. Prior to testing, the cells on the sample surfaces were rinsed with phosphate‐buffered saline (PBS). The optical density (OD) value was then measured at a wavelength of 450 nm using a spectrophotometer (SpectraMax iD3, Molecular Devices, USA). Additionally, the cells were stained using the Live/Dead BacLight bacterial viability kit (Invitrogen, USA) with live and dead cells exhibiting green and red fluorescence, respectively. The staining results were finally observed using a fluorescence microscope (Axio Observer A1, Carl Zeiss, German).

### 4.5 Cyclic Voltammetry (CV) and Electrochemical Impedance Spectroscopy

Cyclic voltammetry (CV) was carried out with a CHI760E Evoltammetric potentiostat in a three‐electrode configuration where the working electrode was a glassy carbon electrode, the counter electrode was a platinum wire, and the pseudo‐reference was an Ag/AgCl wire that was calibrated against ferrocene/ferrocenium redox (Fc/Fc^+^). Cyclic voltammograms for the D‐A copolymer films deposited on the glassy carbon working electrode in CH_3_CN solution containing Bu_4_NPF_6_ (0.1 mol L^−1^) electrolyte at a scan rate of 50 mV s^−1^.

Electrochemical impedance spectroscopy was also carried out using a CHI760E Evoltammetric potentiostat in a three‐electrode configuration, where the working electrode was a monocrystalline silicon evaporated with 3 nm Cr, 50 nm Au, and the material spun on the Au sheet, the counter electrode was a platinum sheet, and the reference electrode was Ag/AgCl. Electrochemical impedance spectroscopy was performed on the polymer films in 0.1 m NaCl electrolyte solution at a scan rate of 50 mV s^−1^.

### 4.6 Grazing‐Incidence Wide‐Angle X‐ray Diffraction (GIWAXS)

GIWAXS experiments were carried out on a Xeuss 3.0 UHR system from Xenocs. The instrument was equipped with a Eiger 1M detector with a pixel size of 75 µm × 75 µm. The X‐ray source was a Microfocus Sealed Tube X‐ray Cu‐source. The wavelength used was λ = 1.54 Å. The samples were placed vertically on the goniometer at a grazing angle of 0.2° relative to the incident beam. The sample to detector distance (SDD) was 75 mm. The accumulation time for each measurement was 20 min.

### 4.7 Electrochemical Spectra

The equipment used was a UV–vis–NIR spectrophotometer, model LAMBDA 1050+. The experimental procedure was as follows. A 40 µL volume of the prepared 5 mg/mL PDPPXO‐2TzC4 solution was spin‐coated onto an ITO glass substrate and annealed at 100°C for 30 min. The ITO glass was placed in a cuvette containing a 0.1 m NaCl solution. The working electrode of the electrochemical workstation was connected to the ITO, with an Ag/AgCl electrode serving as the reference and counter electrode. Prior to testing, a 60‐s pre‐doping step was conducted, followed by spectral scanning. The scanning voltage range was 0 to 1.2 V, and the wavelength range was 1400 to 300 nm.

### 4.8 Atomic Force Microscopy (AFM)

The equipment used was an Atomic Force Microscope with model Dimension FastScan, probe type FMV‐A, and data analysis performed using NanoScope Analysis 3.00 (Bruker Nano Analytics). The experimental procedure was as follows: An 8 µL aliquot of the prepared 5 mg/mL PDPPXO‐2TzC4 solution was taken and spin‐coated onto the glass surface, followed by annealing at 100°C for 30 min. AFM measurements were performed in contact mode with a scan size set to 3 µm, and the tests were conducted at a room temperature of approximately 25°C.

### 4.9 Water Contact Angle

Water contact angle measurements were performed using a DSA 100S contact angle goniometer. First, 40 µL of the pre‐prepared 5 mg/mL PDPPXO‐2TzC4 solution was spin‐coated onto a 2×2 cm^2^ glass substrate, followed by annealing at 100°C for 30 min. The glass slide with the polymer thin film was placed on the horizontal stage of the contact angle goniometer. Three uniform‐sized droplets of ultrapure water were dispensed onto the film. Contact angle measurements were performed for each droplet, and the final value was calculated as the average of the three measurements.

## Conflicts of Interest

The authors declare no conflict of interest.

## Supporting information




**Supporting File**: advs73927‐sup‐0001‐SuppMat.docx.

## Data Availability

The data that support the findings of this study are available from the corresponding author upon reasonable request.
