# Supplementary material for: High‐Performance Ambipolar Organic Electrochemical Transistors Based on Diketopyrrolopyrrole‐Dialkoxybithiazole Conjugated Polymers for Single‐component Inverters
Source: Adv Sci (Weinh). 2026 Jan 21;13(18):e20003. doi: 10.1002/advs.202520003 (PMC13042758; doi:10.1002/advs.202520003)
Supplement: Supplementary file 1 — Supporting File: advs73927‐sup‐0001‐SuppMat.docx. [file ADVS-13-e20003-s001.docx]

Supporting Information

**High-Performance Ambipolar Organic Electrochemical Transistors Based on Diketopyrrolopyrrole-Dialkoxybithiazole Conjugated Polymers for Single-component Inverter**

Jiazheng Li^1, †^, Zhi Li^1, †^, Jiayu Huang^1, †^, Jingjing Su^3, †^, Peijie Xu^1^, Siwen Wang^1^, Ping Zhang^4^, Yuchuan Tian^5^, Tao Pan^1^, Junyang Liu^1^, Junyu Li^5^, Gang Ye^2,^ *, Ryan C. Chiechi^6,^ *, Yanxi Zhang^1,^ *, Wenjing Hong^1,^ *

Jiazheng Li, Zhi Li, Jiayu Huang, Peijie Xu, Siwen Wang, Tao Pan, Junyang Liu, Yanxi Zhang, Wenjing Hong

^1^Institute of Flexible Electronics (IFE, Future Technologies), State Key Laboratory of Physical Chemistry of Solid Surfaces & IKKEM, College of Chemistry and Chemical Engineering, Xiamen University, Xiamen 361005, China.

**E-mail**: [ifeyxzhang@xmu.edu.cn](mailto:ifeyxzhang@xmu.edu.cn), whong@xmu.edu.cn

Gang Ye

^2^Ministry of Education Key Laboratory for the Green Preparation and Application of Functional Materials, Hubei Key Laboratory of Polymer Materials, School of Materials Science and Engineering, Hubei University, Wuhan 430062, China.

**E-mail:** [g.ye0612@hubu.edu.cn](mailto:g.ye0612@hubu.edu.cn)

Jingjing Su

^3^Stomatological Hospital of Xiamen Medical College, Xiamen Key Laboratory of Stomatological Disease Diagnosis and Treatment, Xiamen 361008, China.

Ping Zhang

^4^School of Electrical Engineering and Automation, Jiangxi University of Science and Technology, Ganzhou, Jiangxi, 341000, China.

Yuchuan Tian, Junyu Li

^5^Sinopec Shanghai Research Institute of Petrochemical Technology, Shanghai 201028, China.

Ryan C. Chiechi

^6^Department of Chemistry & Organic and Carbon Electronics Cluster North Carolina State University Raleigh, NC 27695-8204, USA.

**E-mail:** ryan.chiechi@ncsu.edu

† These authors contributed equally.

**Table of Contents**

**1. Synthesis and Characterization of materials**

**2. Supplementary Figures and Tables**

**3. References**

**1. Synthesis and Characterization of materials**

**1.1 Reagents**

All reagents and solvents were commercial and were used as received. 4,4'-dibutoxy-2,2'-bis(trimethylstannyl)-5,5'-bithiazole (**2Tz-C4**), 3,6-bis(5-bromothiophen-2-yl)-2,5-bis(2-(2-(2-methoxyethoxy)ethoxy)ethyl)-2,5-dihydropyrrolo[3,4-c]pyrrole-1,4-dione (**DPP-3O-br**) and 3,6-bis(5-bromothiophen-2-yl)-2,5-di(2,5,8,11,14-pentaoxahexadecan-16-yl)-2,5-dihydropyrrolo[3,4-c]pyrrole-1,4-dione (**DPP-5O-br**) were purchased from Suna Tech. Inc. 1,1,1,3,3,3-Hexafluoro-2-propanol (HFIP), etonitrile were purchased from Shanghai Acmec Biochemical Technology Co., Ltd

**1.2 Characterization**

GPC tests were conducted on a Waters ACQUITY APC room-temperature GPC/SEC system, with the test temperature set at 35 ℃. Hexafluoroisopropanol (HFIP) was used as the eluent, and polystyrene standards were employed for calibration and comparison.

**1.3 General Synthetic Procedures for the DPP based Polymers**

To a dry three-neck flask, dibromo DPP-based monomer (0.1 mmol) and distannyl dialkoxybithiazole-based monomer (0.1 mmol) were added under argon followed by tris(dibenzylideneacetone) dipalladium [Pd_2_(dba)_3_] (6.4 mg) and tri(o-tolyl)phosphine [P(o-tolyl)_3_] (9.6 mg). The flask and its contents were subjected to 3 pump/purge cycles with N_2_ followed by addition of anhydrous, degassed chlorobenzene (5 mL) via syringe. The reaction mixture was stirred at 120 ℃ for three days. After cooling to room temperature, the deeply green colored reaction mixture was dropped into 100 mL vigorously stirred methanol (containing 5 mL 12 M hydrochloride acid). After stirring for 4 hours, the precipitated solid was collected by filtration. The solid polymers were re-dissolved in chloroform and reprecipitated into methanol. After filtration, the polymers were subjected to sequential Soxhlet extraction. The sequential solvents were methanol, hexane and chloroform or hexafluoropropanol. Impurities and low-molecular-weight fractions were removed by methanol and hexane. Finally, the polymer solution in chloroform for PDPP5O-2TzC4 or hexafluoropropanol for PDPP3O-2TzC4 was concentrated to give the polymers as dark solid.

**Figure S1.** Synthetic route for PDPP3O-2TzC4 and PDPP5O-2TzC4.

**PDPP3O-2TzC4**: Synthesis according to the general polymerization procedure: monomer DPP3O-Br (75 mg, 0.1 mmol), monomer 2TzC4 (63.8 mg, 0.1 mmol), dry chlorobenzene (5 mL). The target polymer was obtained as a dark solid (96 mg, 92 %). GPC: Mn =12.7 kDa, Mw =43.2 kDa, PDI =3.38.

**PDPP5O-2TzC4**: Synthesis according to the general polymerization procedure: monomer DPP5O-Br (93 mg, 0.1 mmol), monomer 2TzC4 (63.8 mg, 0.1 mmol), dry chlorobenzene (5 mL). The target polymer was obtained as a dark solid (96 mg, 92 %). GPC: Mn =11.7 kDa, Mw =34.0 kDa, PDI =2.89.

**1.4 Density Functional Theory (DFT) and Complete Active Space Self-Consistent Field (CASSCF) Calculations**

To model and contexualize the electronic structure of PDPP3O-2TzC4 and PDPP5O-2TzC4, performed calculations on the trimer of their common backbone with the side-chains truncated. For reference, we also calculated the analogous backbone with thiophenes (Th) in place of the thiazoles (Tz), i.e. a Th-DPP-Th-Th-Th backbone where Th is thiophene. We minimizes the gas-phase geometries and computed the single-point energies using ωB97M-D4/Def2TZVP DFT.^[1-2]^ This combination of range-separated double hybrid functional and triplezeta basis set eliminates the systematic errors of B3LYP/6-31G that are particularly acute for extended conjugation.^[3]^ The results in **Figure S2** show that both backbones are planar with highly delocalized frontier molecular orbitals. Compared to the all-thiophene analog, the inclusion of thiazole rings pulls the LUMO down by ~200 meV and pushes the HOMO up by ~500 meV, predicting that PDPP3O-2TzC4 and PDPP5O-2TzC4 are good acceptors (n-type polymers) and have narrow band gaps, in agreement with the experimental data.


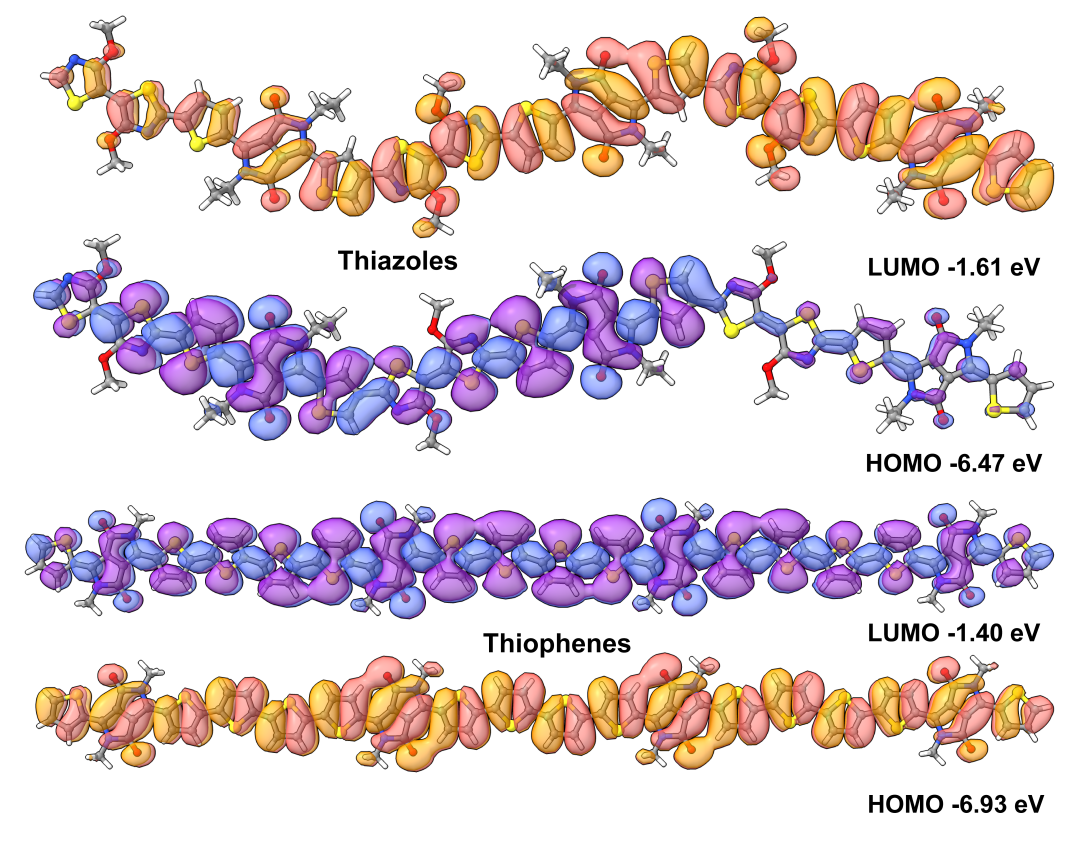


**Figure S2.** Isoplots (0.005 au) of frontier molecular orbitals of the trimer of the common backbone of PDPP3O-2TzC4 and PDPP5O-2TzC4 (top pair) and the all-thiophene analog (bottom pair) computed using ωB97M-D4/Def2-TZVP DFT.

End-group effects are more prevalent for the PDPP3O-2TzC4 and PDPP5O-2TzC4 backbones than the thiophene analogs, suggesting that the trimer might not be sufficient to model the persistence conjugation length. We therefore modeled the hexamer using the same methodology as we used to find the gas-phase minimized geometry of the trimer, but using the much more computationally efficient GFN2-xTB for the geometry optimization step.^[4]^ The results are shown in **Figure S3**. Although the end-group effects persist, the apparent spatial separation of the HOMO and LUMO vanishes in the hexamer. The orbital energies are unchanged, suggesting that both trimer and hexamer are sufficient to model the conjugation length appropriately. However, inspection of the nearby orbitals shows that the three occupied and unoccupied states at the frontier states are all within ~200 meV with the symmetry as the frontier orbitals. The HOMO-1 and LUMO+1 shown in **Figure S3** strongly suggest (near)degeneracy that single-determinate DFT is unable to describe correctly. This artificial separation of the π-π* states can be predictive of the presence of highly correlated ground-state electrons, which could explain the observation of an EPR signal at room temperature. We computed the degree of spin contamination using methods described elsewhere to predict open-shell ground-states,^[5-7]^ but none was found for the trimer or the hexamer.


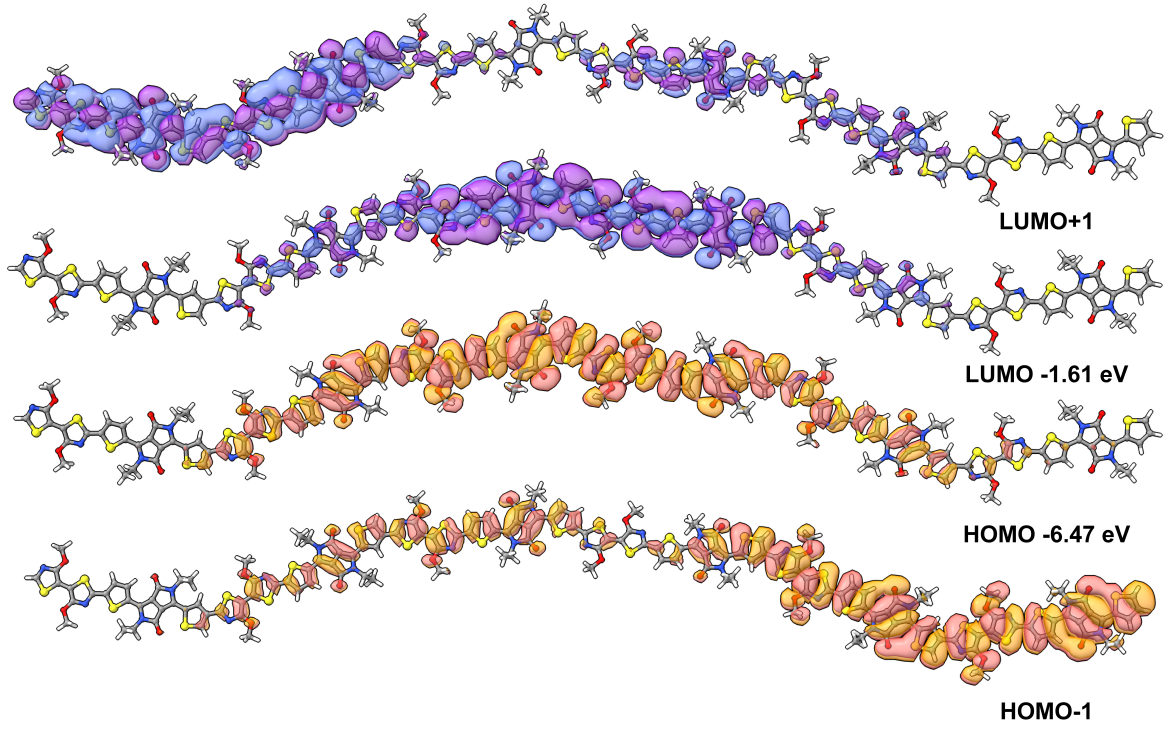


**Figure S3.** Isoplots (0.005 au) of frontier molecular orbitals of the hexamer of the common backbone of PDPP3O-2TzC4 and PDPP5O-2TzC4 computed using ωB97M-D4/Def2-TZVP DFT. Both orbitals show excellent delocalization in the backbone and end-group effects that are artifacts of using oligomers to approximate a polymer structure.

The observation of an EPR signal at room temperature indicates a high-spin state driven by the acceptor-acceptor topology and small band-gap. One possibility is a thermally accessible triplet state, but DFT predicts a singlet-triplet gap of 1.44 eV, which is not accessible at room temperature. This value, in combination with the results above, indicate that single-determinate DFT is unable to describe this electronic structure correctly. We therefore performed a singlet CASSCF(12,10) on the model trimer backbone. We used the formula developed by Head-Gordon^[8]^ to compute **$N_{U}=\sum_{i=1}^{\text{m}} \text{ n}_{\text{i}}^{2}(2-\text{n}_{\text{i}})^{2}\approx0.93$** where a closed-shell system would yield *N*_U_ = 0 and a pure diradical would yield *N*_U_ = 2. The value of *N*_U_ ≈ 0*.*93 corresponds to static electron correlation in which occupied and unoccupied frontier states mix, leading to diradical character. **Figure S4** shows isoplots of three CASSCF natural orbitals with nonzero occupancy. The red arrows indicate density in non-bonding p-orbitals, which if fully populated would describe the diradical Lewis structure shown in **Figure S4b**.


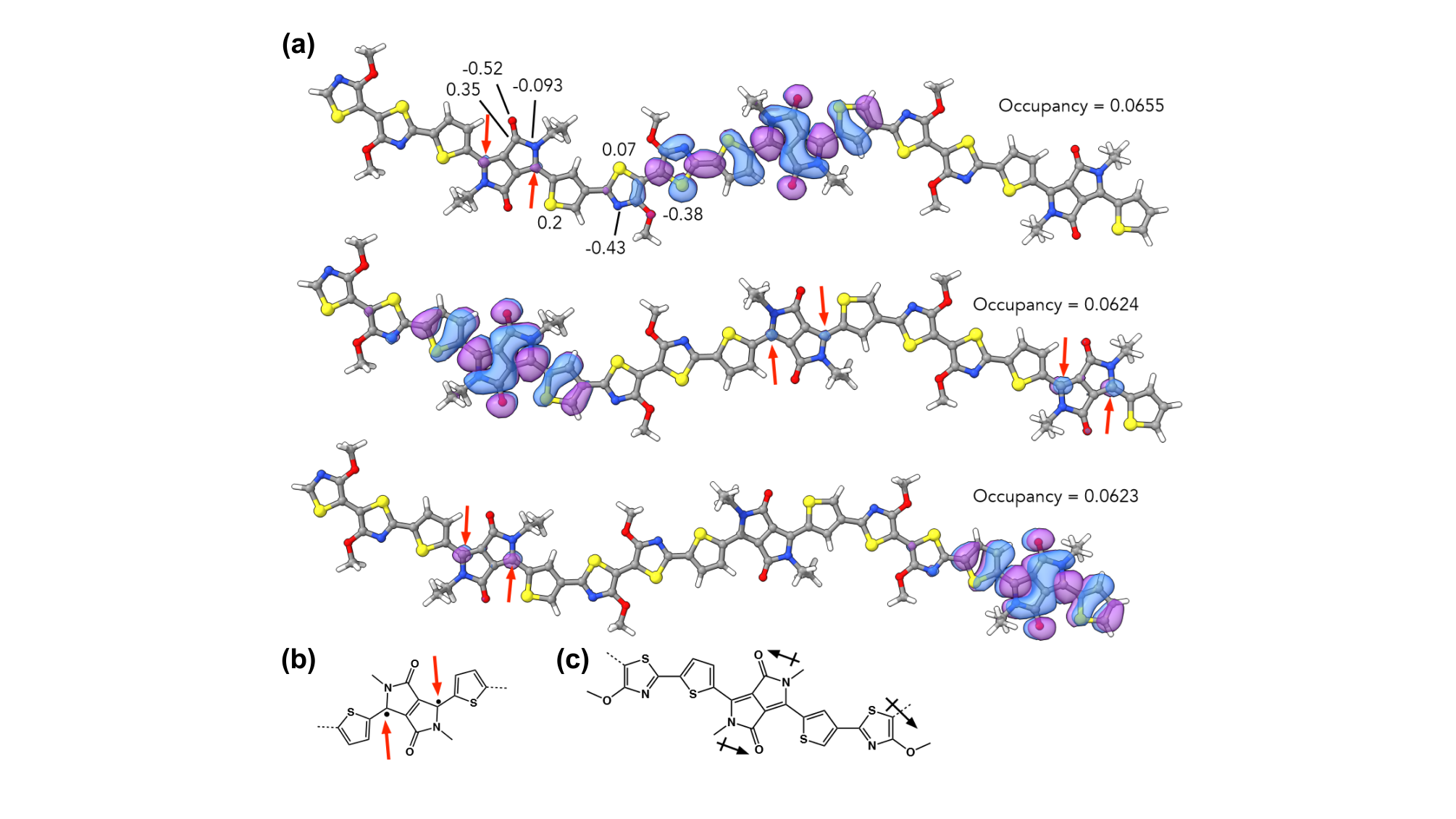


**Figure S4.** a) Isoplots (0.005au) of three CASSCF natural orbitals of the trimers of the common backbone of PDPP3O-2TzC4 and PDPP5O-2TzC4 computed using ωB97M-D4. The numbers are Mulliken charges. The red arrows indicate contributions to the ground-state electronic structure from anti-bonding p-orbitals. b) The Lewis structure of the diradical character from the nonzero occupancy of the p-orbitals. c) The Lewis structure of the close-shell character showing the dipole moments from the acceptor-acceptor structure.

**2. Supplementary Figures and Tables**


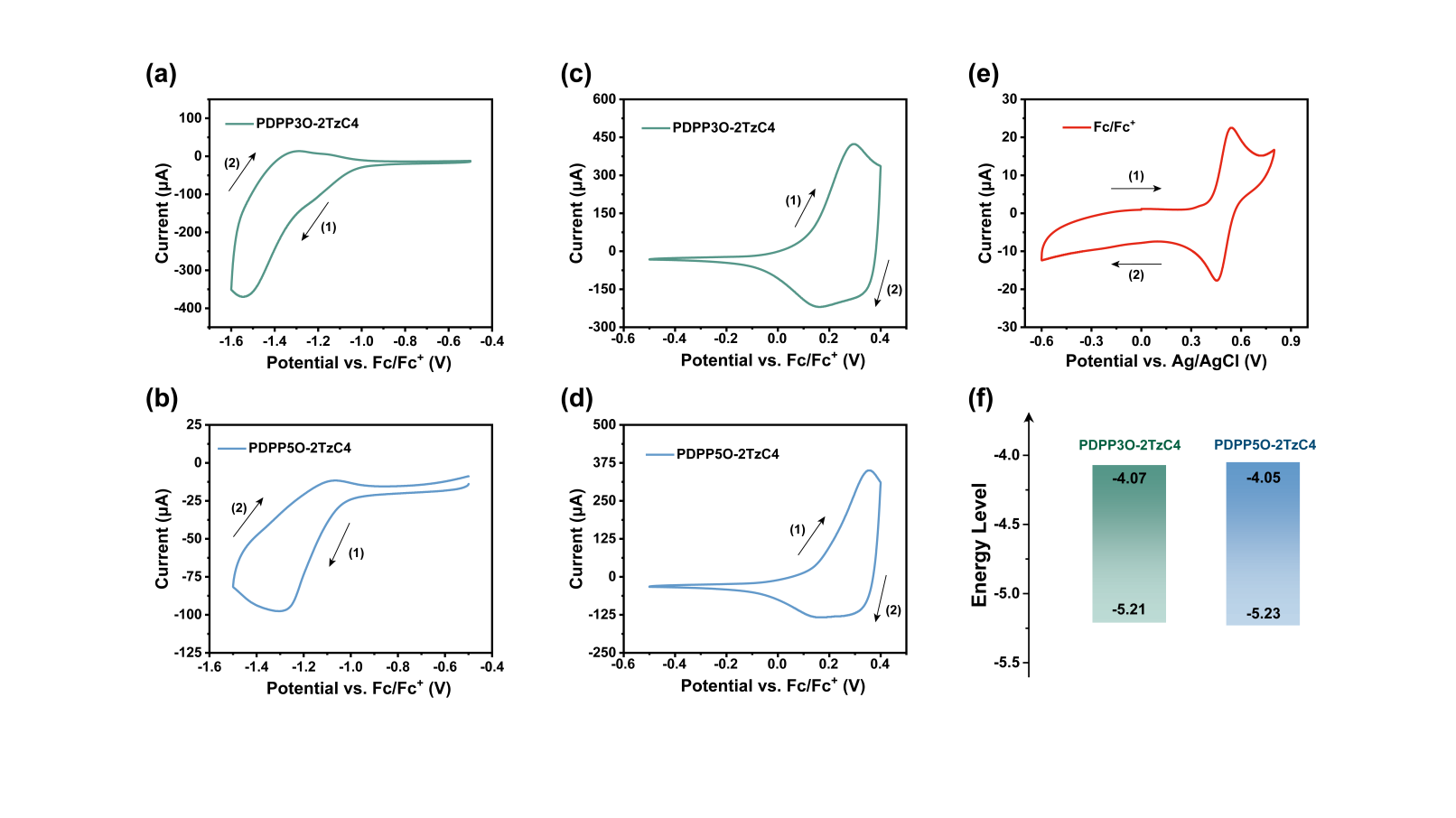


**Figure S5.** a, b, c, d) Cyclic voltammetry (CV) curves of the two polymer films in CH_3_CN (containing 0.1 M Bu_4_NPF_6_) vs. Fc/Fc^+^. e) The CV curve of Fe(C_5_H_5_)_2_ was measured in a CH_3_CN solution (containing 0.1 M Bu_4_NPF_6_), with the half-wave potential of Fe(C_5_H_5_)_2_ being 0.5 V. f) Molecular energy levels of PDPP3O-2TzC4 and PDPP5O-2TzC4.


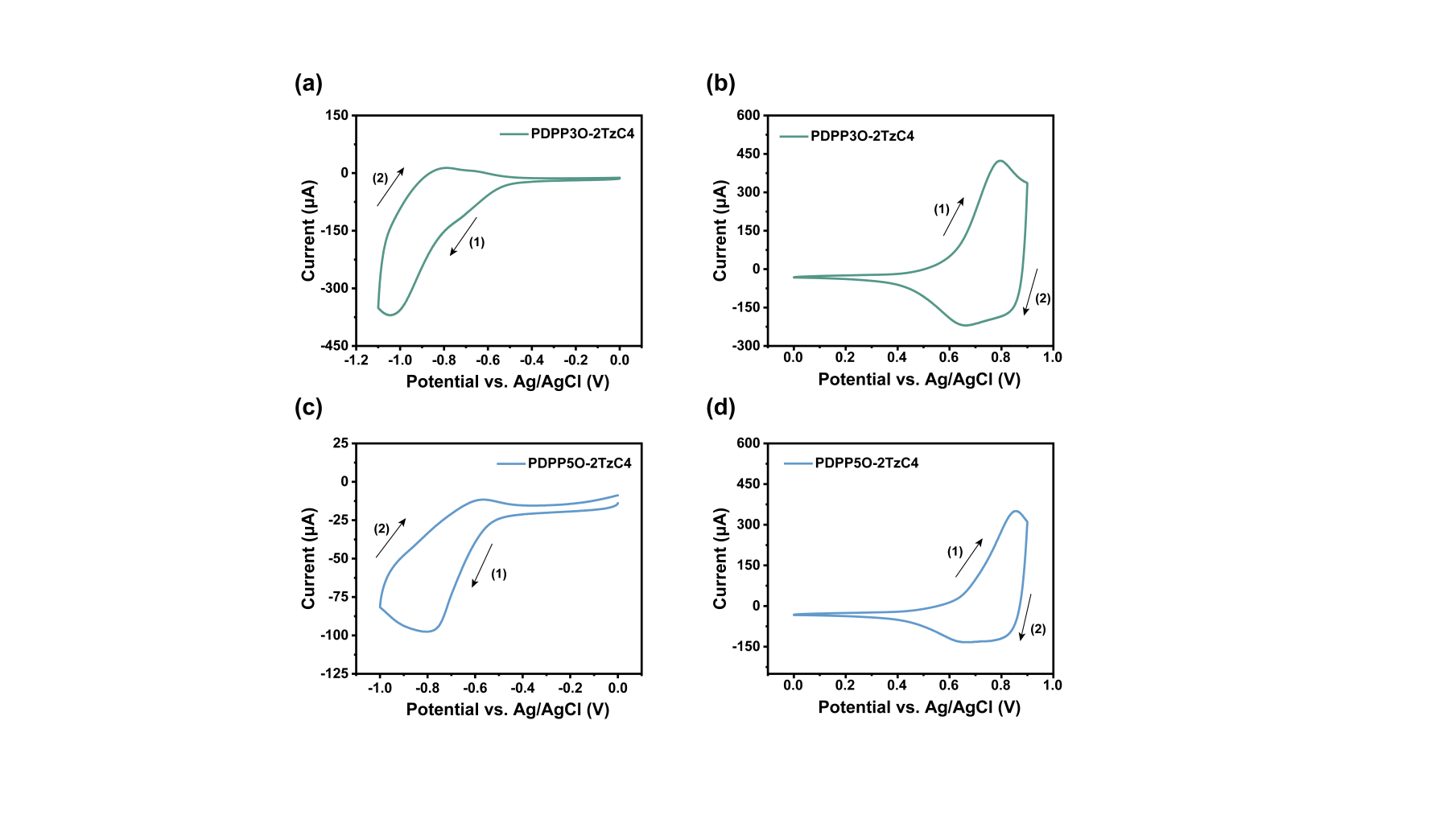


**Figure S6.** CV curves of the PDPP3O-2TzC4 and PDPP5O-2TzC4 films in CH_3_CN (containing 0.1 M Bu_4_NPF_6_) vs. Ag/AgCl.


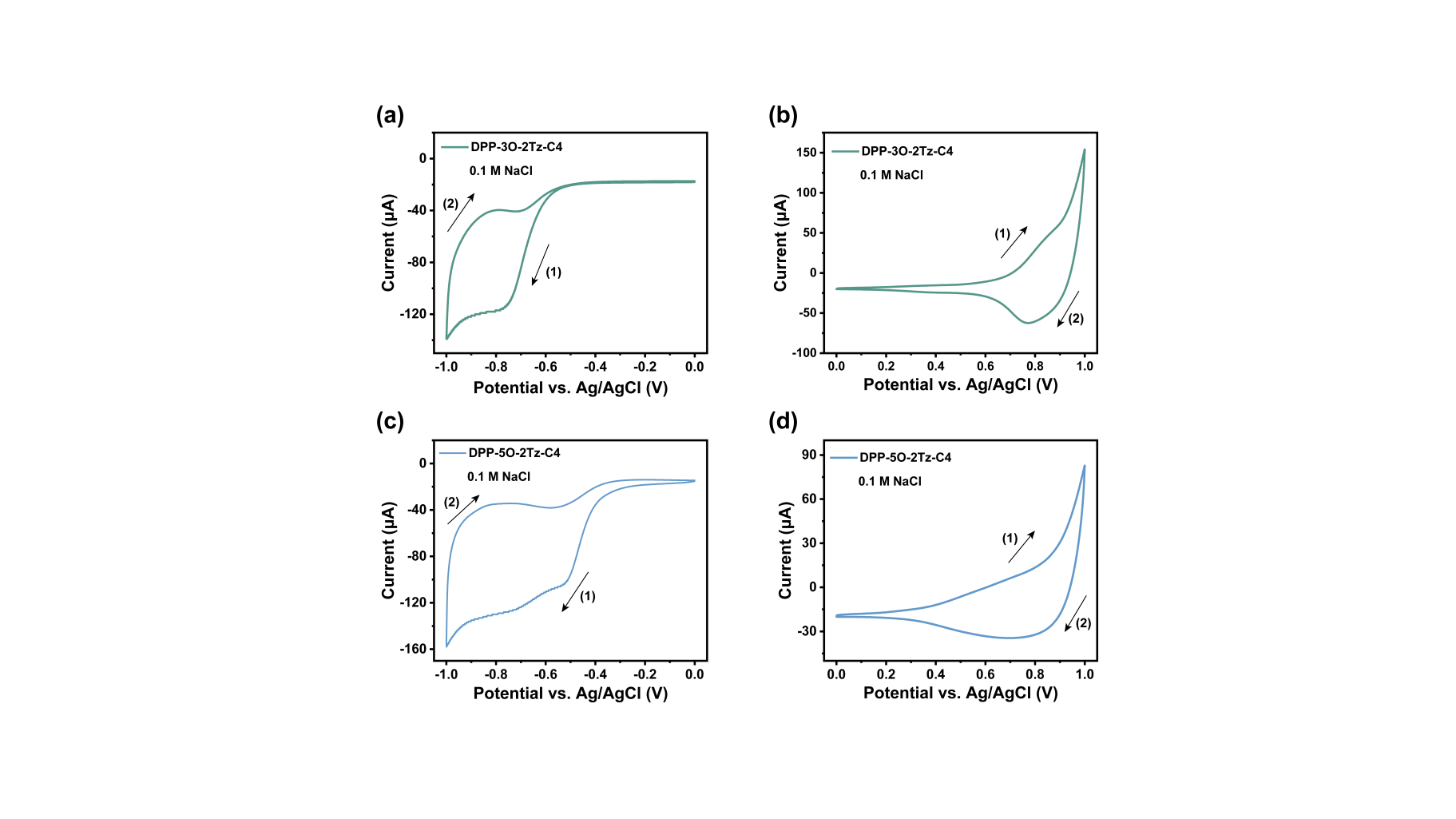


**Figure S7.** CV curves of PDPP3O-2TzC4 and PDPP5O-2TzC4 films in 0.1 M NaCl vs. Ag/AgCl. The CV test was then performed using the ITO/polymer film as the working electrode, an Ag/AgCl reference electrode, a Pt plate counter electrode, and a scan rate of 50 mV/s.


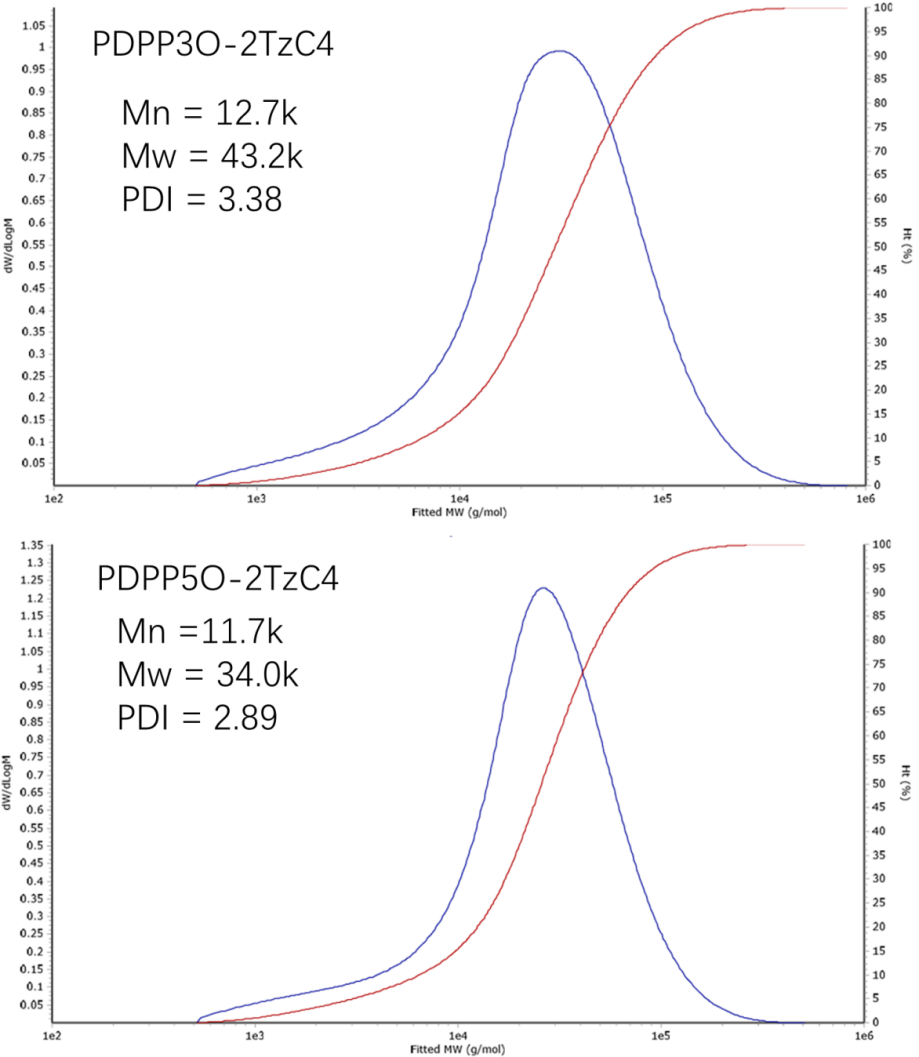


**Figure S8.** GPC of PDPP3O-2TzC4 and PDPP5O-2TzC4.


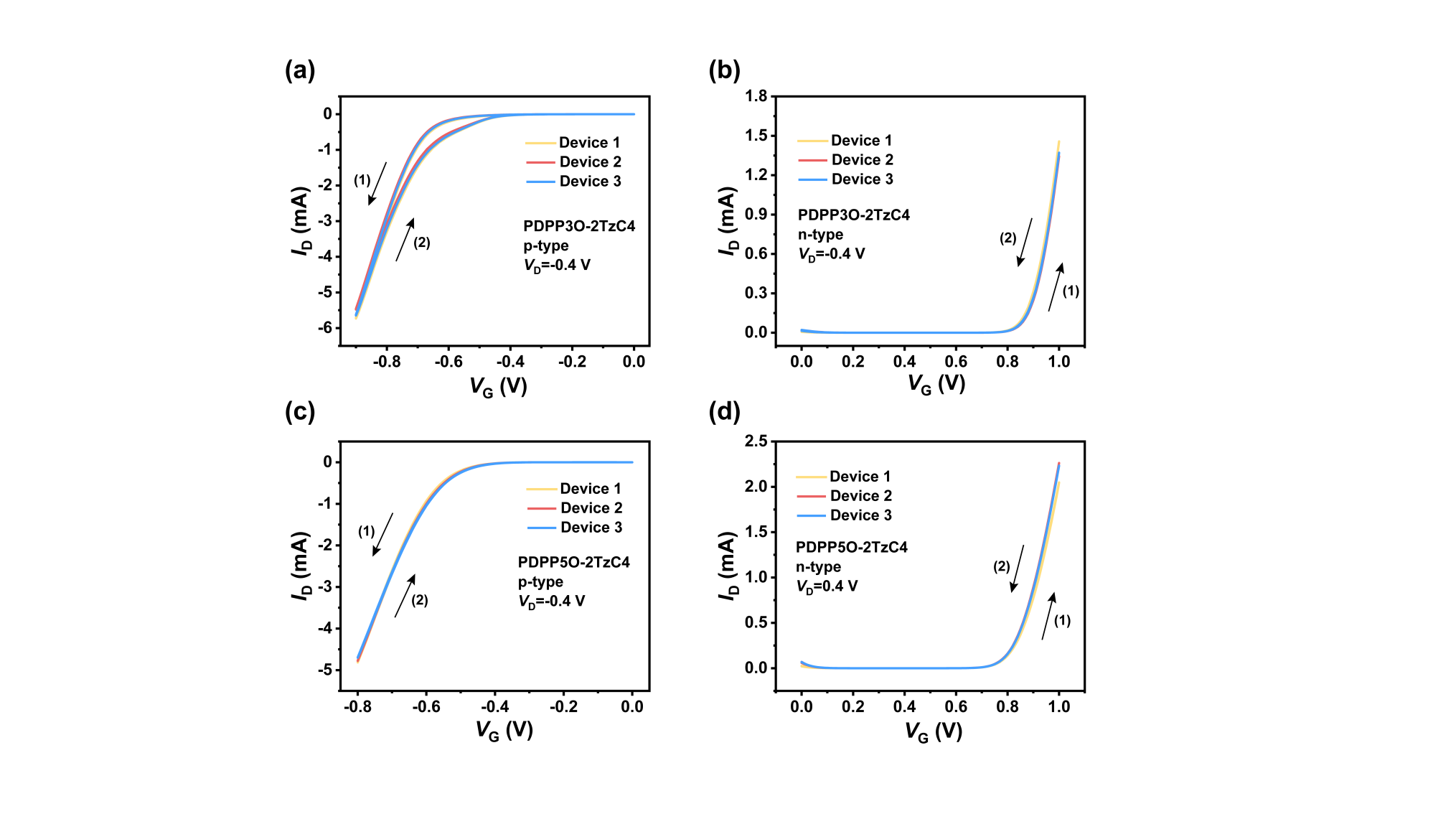


**Figure S9.** Transfer curves obtained from three independent interdigitated electrodes demonstrate excellent device reproducibility, with (a) p-type and (b) n-type characteristics for PDPP3O-2TzC4, and (c) p-type and (d) n-type characteristics for PDPP5O-2TzC4, all including both (1) forward and (2) backward voltage sweeps.


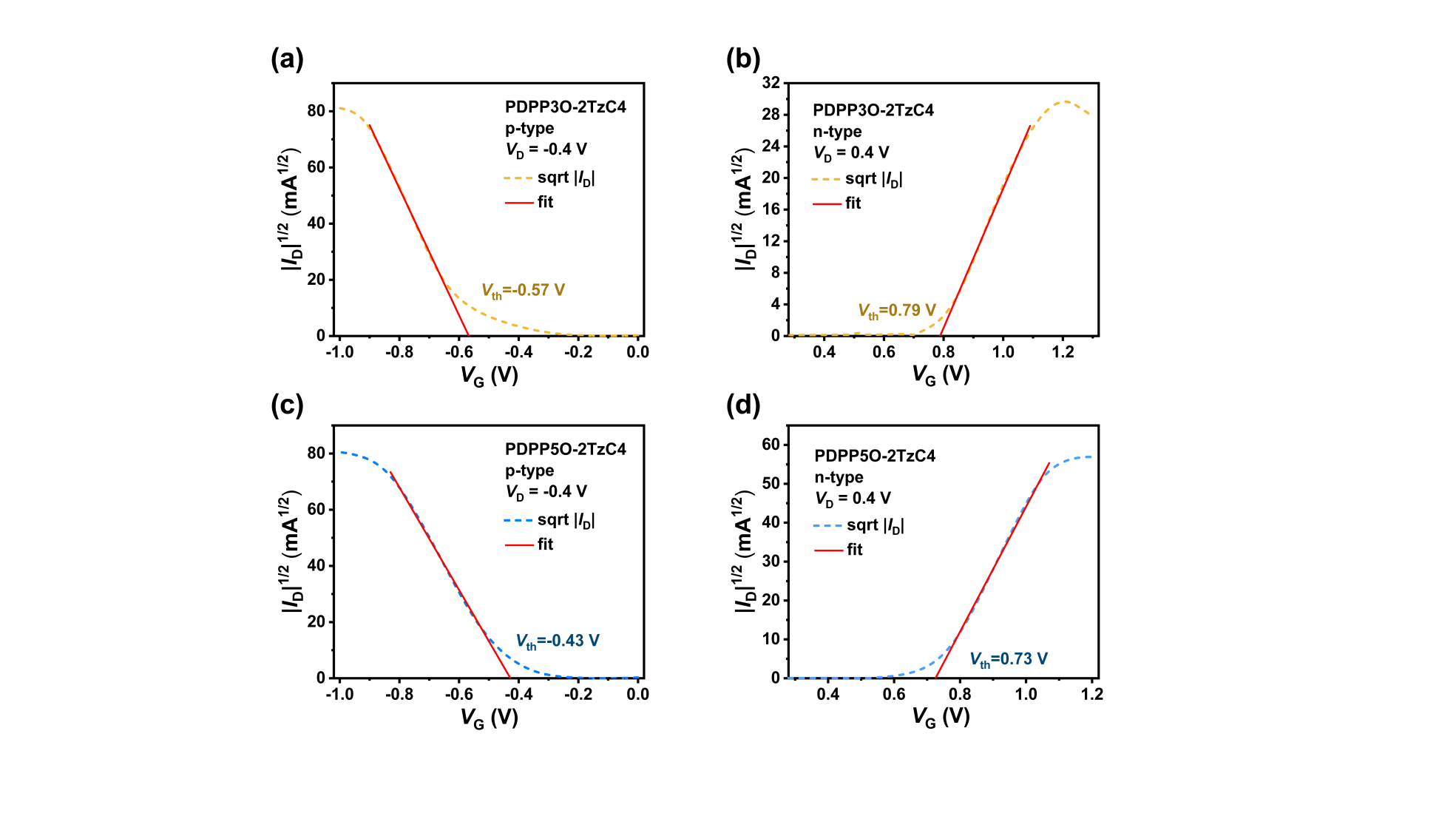


**Figure S10.** a) p-type and b) n-type threshold voltages (*V*_th_) for PDPP3O-2TzC4. c) p-type and d) n-type *V*_th_ for PDPP5O-2TzC4.


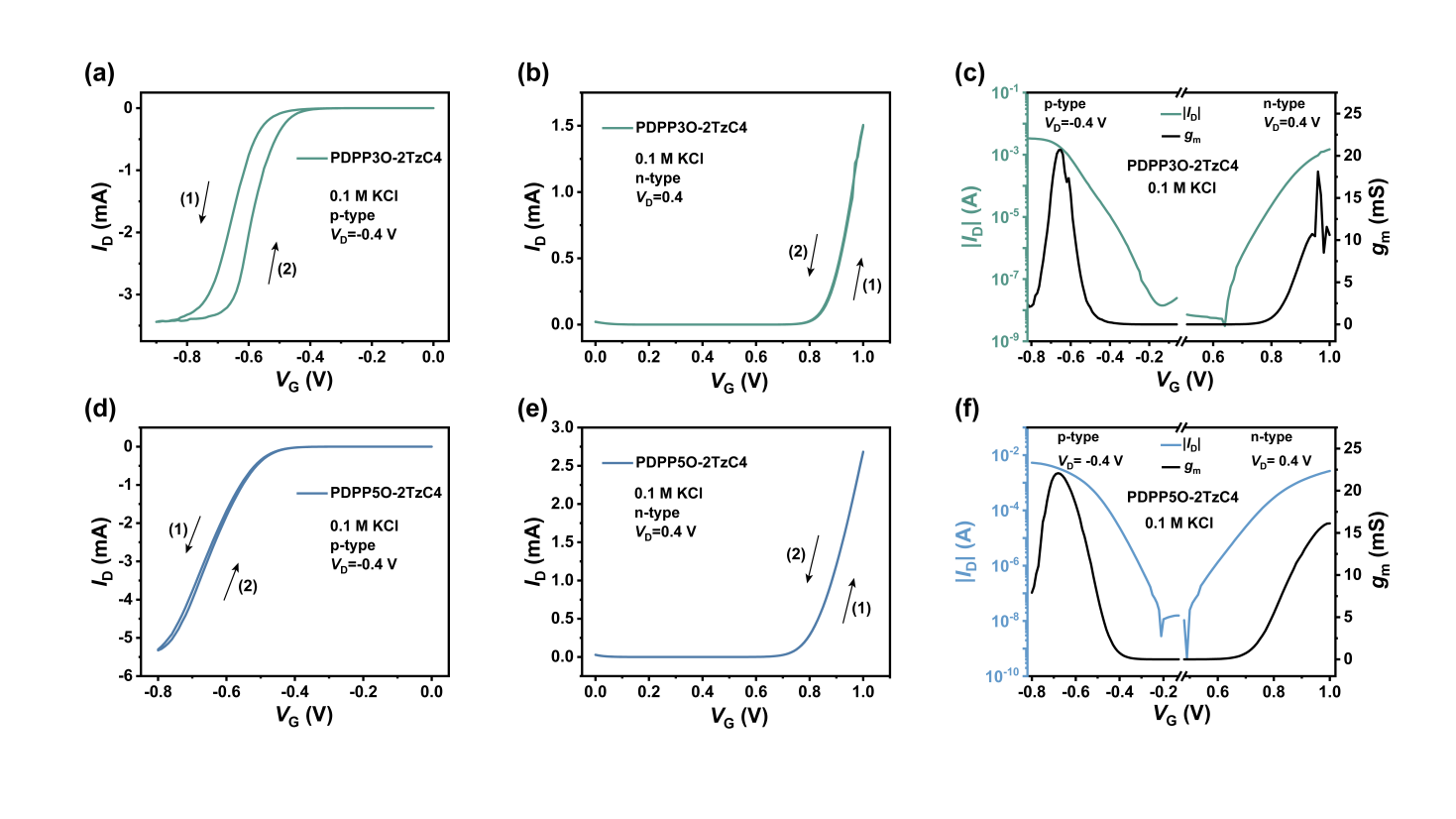


**Figure S11.** (a) p-type and (b) n-type transfer characteristics, and (c) *I*_on/off_ and transconductance curves for PDPP3O-2TzC4; (d) p-type and (e) n-type transfer characteristics, and (f) *I*_on/off_ and transconductance curves for PDPP5O-2TzC4, all measured in 0.1 M KCl electrolyte. Unless otherwise specified, all other devices were characterized using 0.1 M NaCl as the electrolyte.


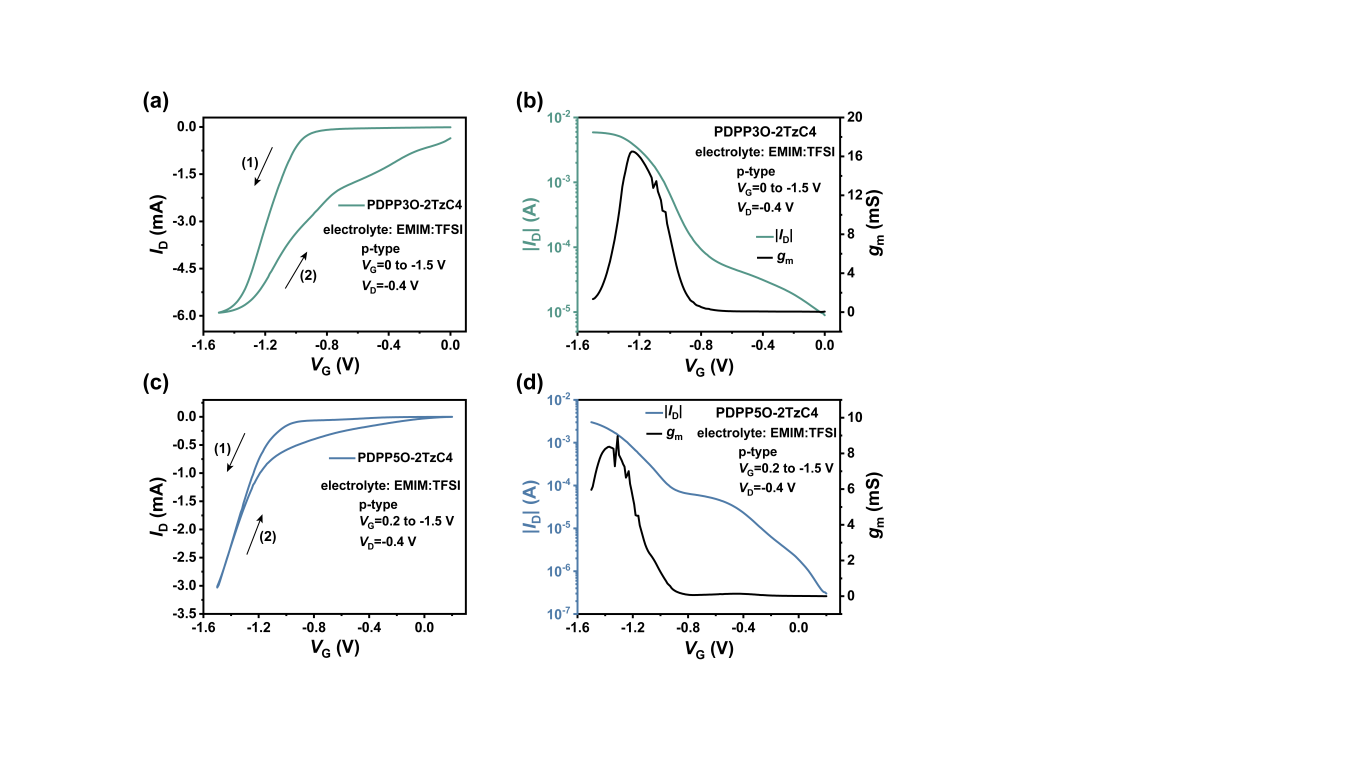


**Figure S12.** a) Transfer curve and b) Switching on-off ratio and transconductance for the OECT device based on PDPP3O-2TzC4 using EMIM:TFSI mixed with P(VDF-HFP) as the solid-state electrolyte. c) Transfer curve and d) Switching on-off ratio and transconductance for the OECT device based on PDPP5O-2TzC4 using EMIM:TFSI mixed with P(VDF-HFP) as the solid-state electrolyte. (1) Forward and (2) Backward voltage sweeps.


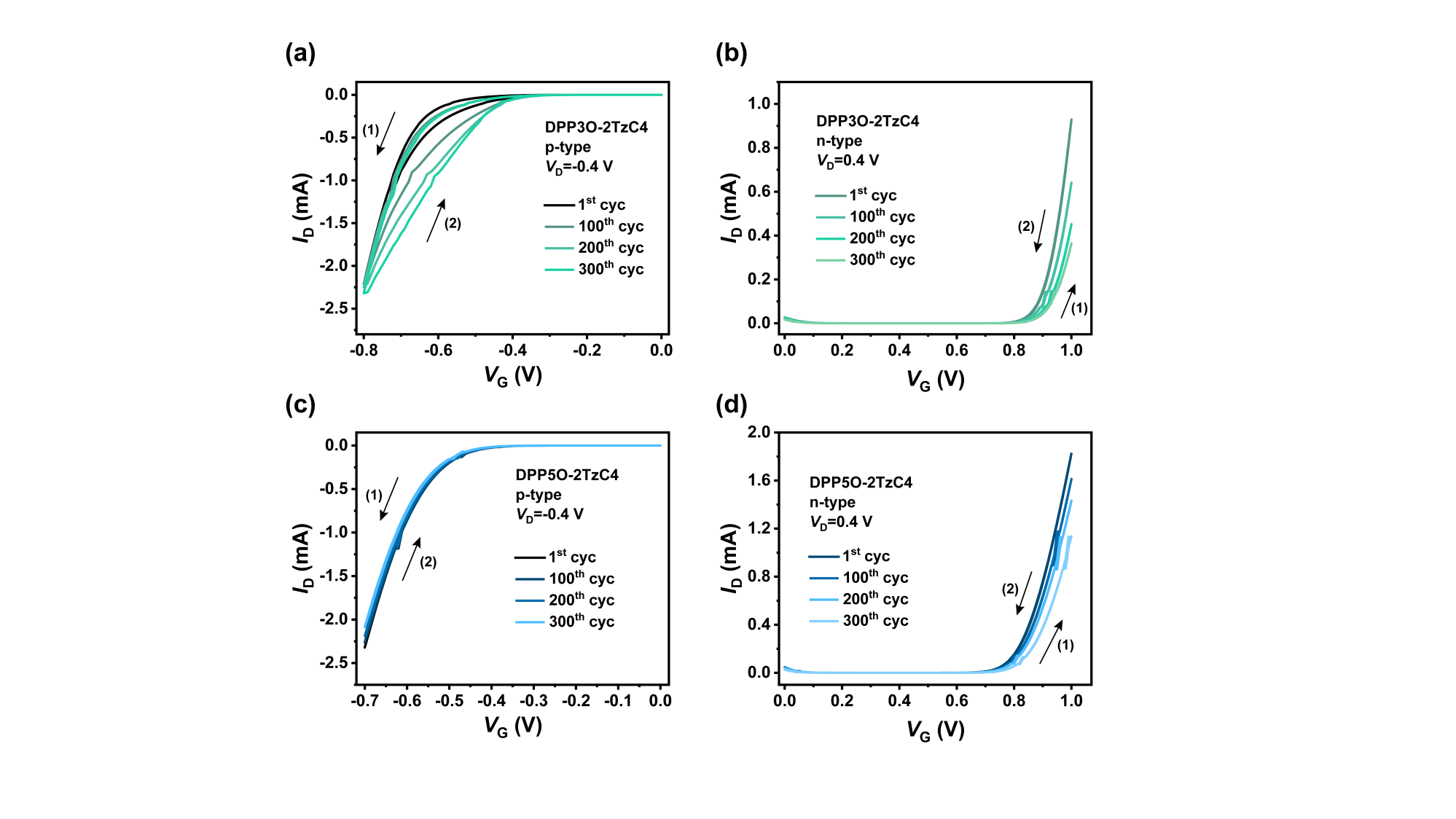


**Figure S13.** a, c) Comparison of the 1^st^, 100^th^, 200^th^, and 300^th^ transfer curve cycles of the p-type of PDPP3O-2TzC4 and PDPP5O-2TzC4 respectively. b, d) Comparison of the 1^st^, 100^th^, 200^th^, and 300^th^ transfer curve cycles of the n-type of PDPP3O-2TzC4 and PDPP5O-2TzC4 respectively. (1) Forward and (2) Backward voltage sweeps. After 300 cycles, the drain current (*I*_D_) for p-type operation of PDPP3O-2TzC4 and PDPP5O-2TzC4 changed by 3.04% and 10.18% respectively, while for n-type operation the *I*_D_ decreased by 60.74% and 37.74% respectively.


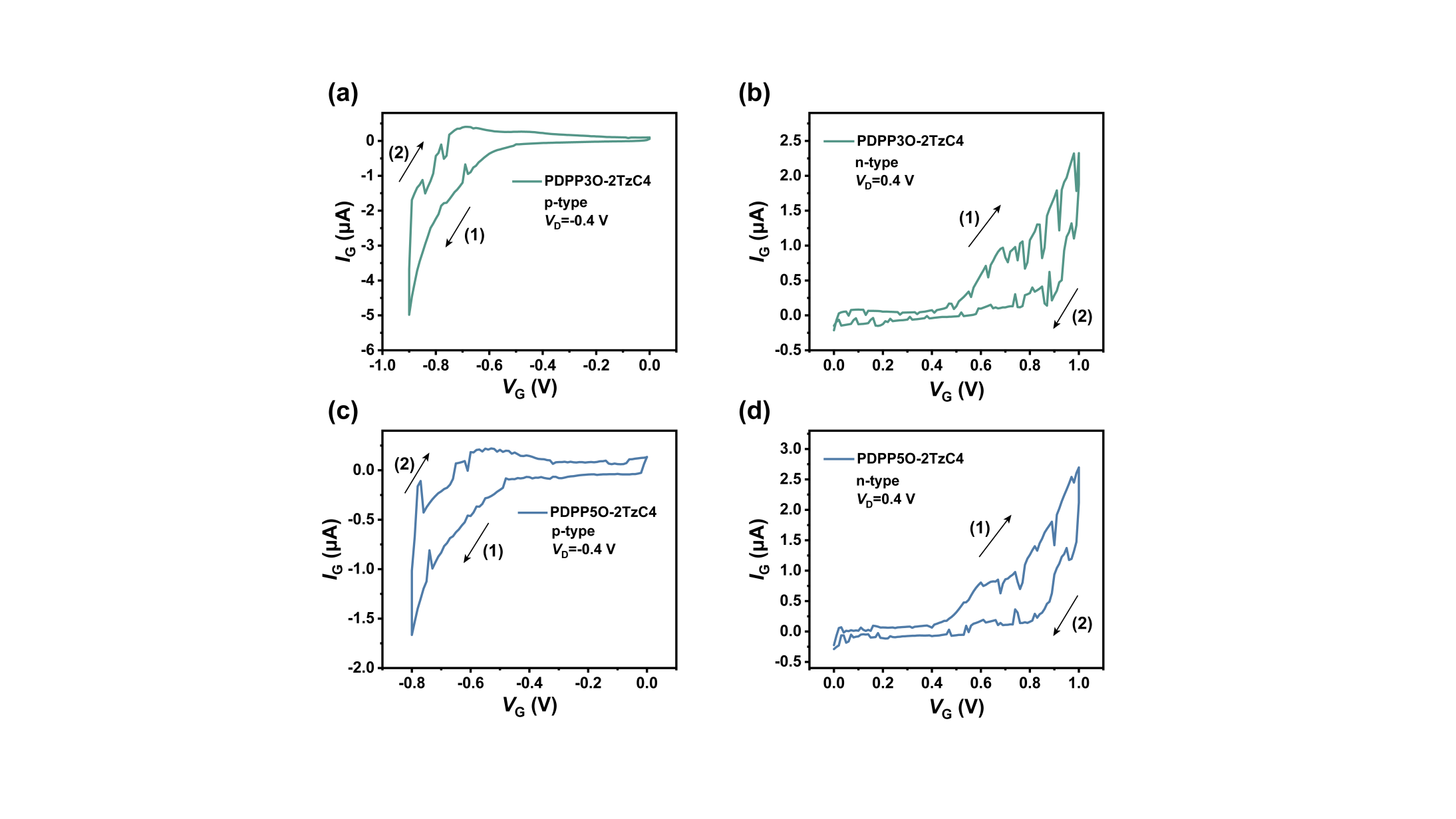


**Figure S14.** a) p-type and b) n-type gate currents (*I*_G_) for PDPP3O-2TzC4. c) p-type and d) n-type *I*_G_ for PDPP5O-2TzC4. (1) Forward and (2) Backward voltage sweeps.


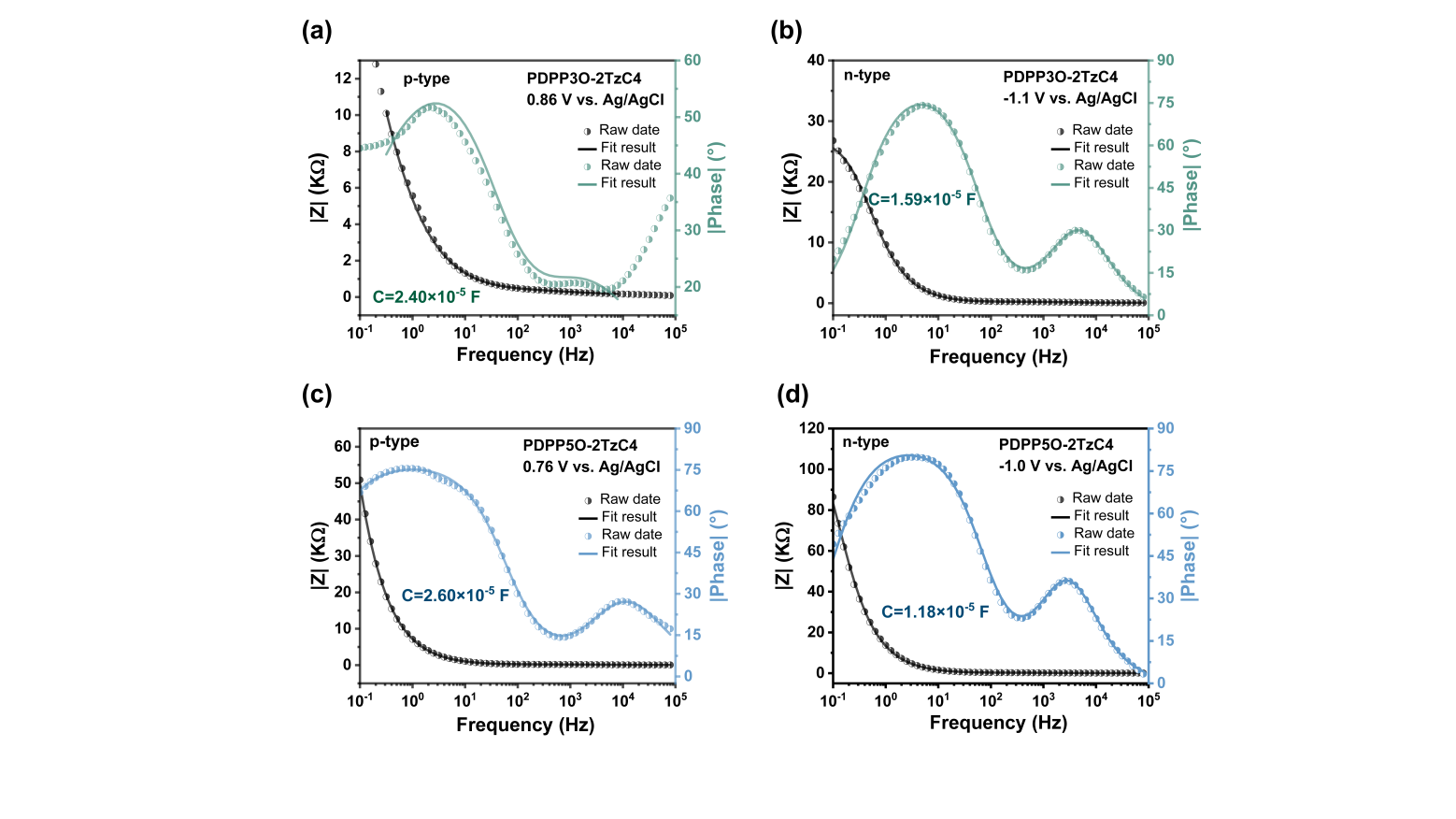


**Figure S15**. The Bode diagrams of PDPP3O-2TzC4 for a) p-type and b) n-type at the *V*_G_ corresponding to the maximum transconductance (*g*_m, max_), and the Bode diagrams of PDPP5O-2TzC4 for c) p-type and d) n-type at the *V*_G_ corresponding to the *g*_m, max_.


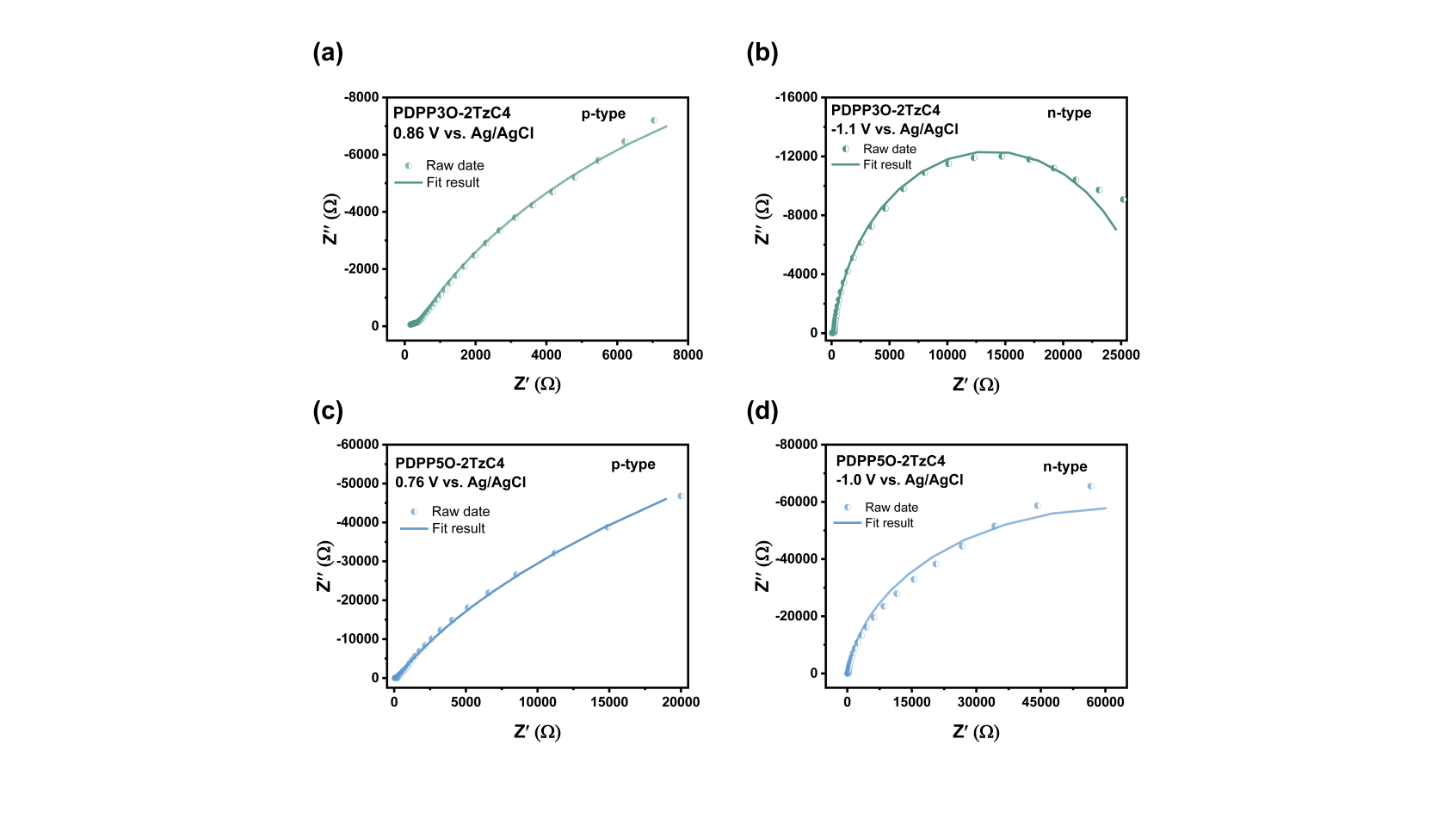


**Figure S16**. The Nyquist diagrams of PDPP3O-2TzC4 for a) p-type and b) n-type at the *V*_G_ corresponding to the *g*_m, max_, and the Nyquist diagrams of PDPP5O-2TzC4 for c) p-type and d) n-type at the *V*_G_ corresponding to the *g*_m, max_.


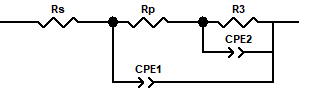


**Figure S17.** Equivalent fitted circuits with double capacitive elements.


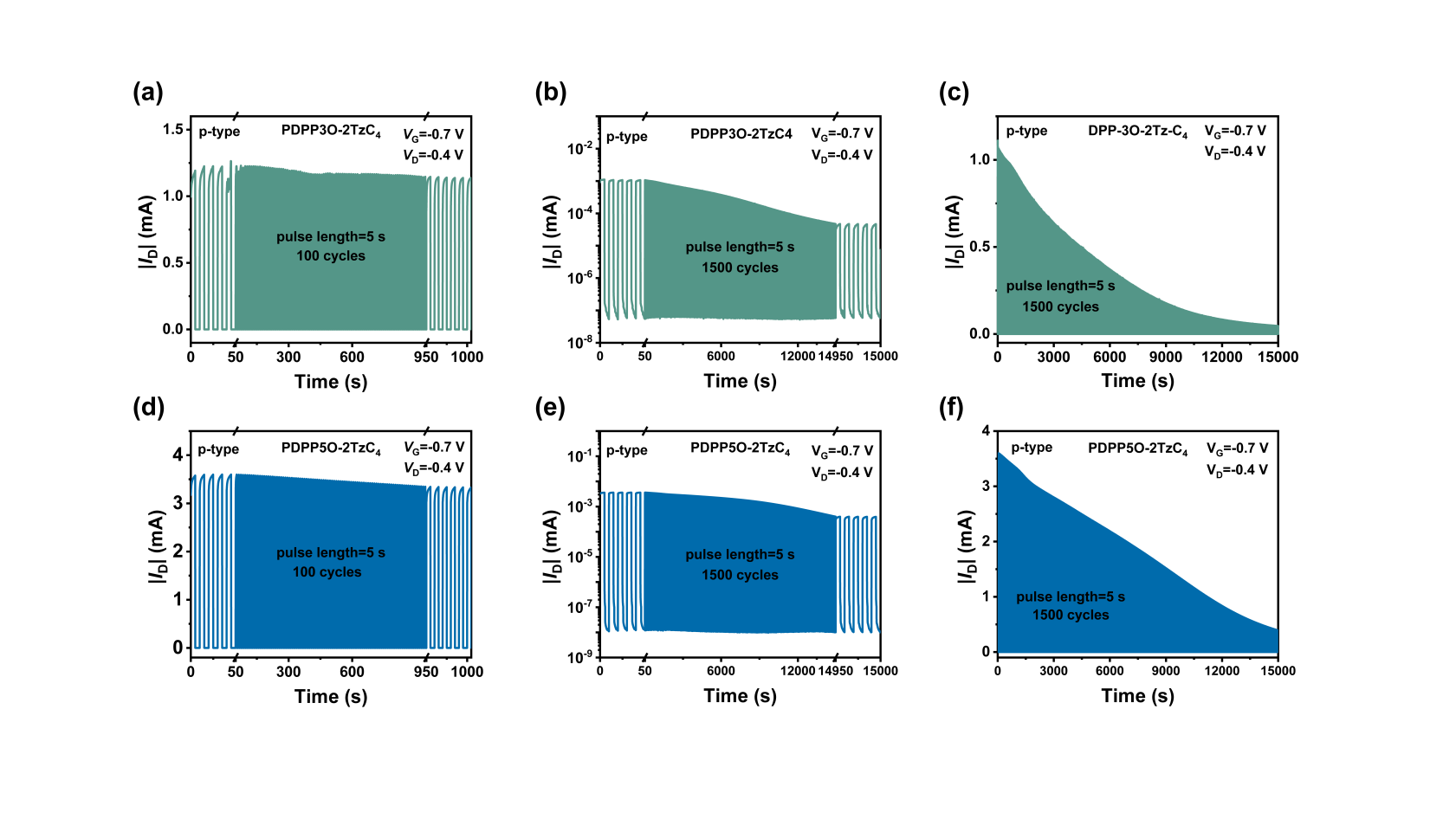


**Figure S18.** **The p-type stability of PDPP3O-2TzC4 and PDPP5O-2TzC4.** Stability of 100 switching cycles for a) PDPP3O-2TzC4 and d) PDPP5O-2TzC4. b, c) Stability of 1500 switching cycles for PDPP3O-2TzC4. e, f) Stability of 1500 switching cycles for PDPP5O-2TzC4.


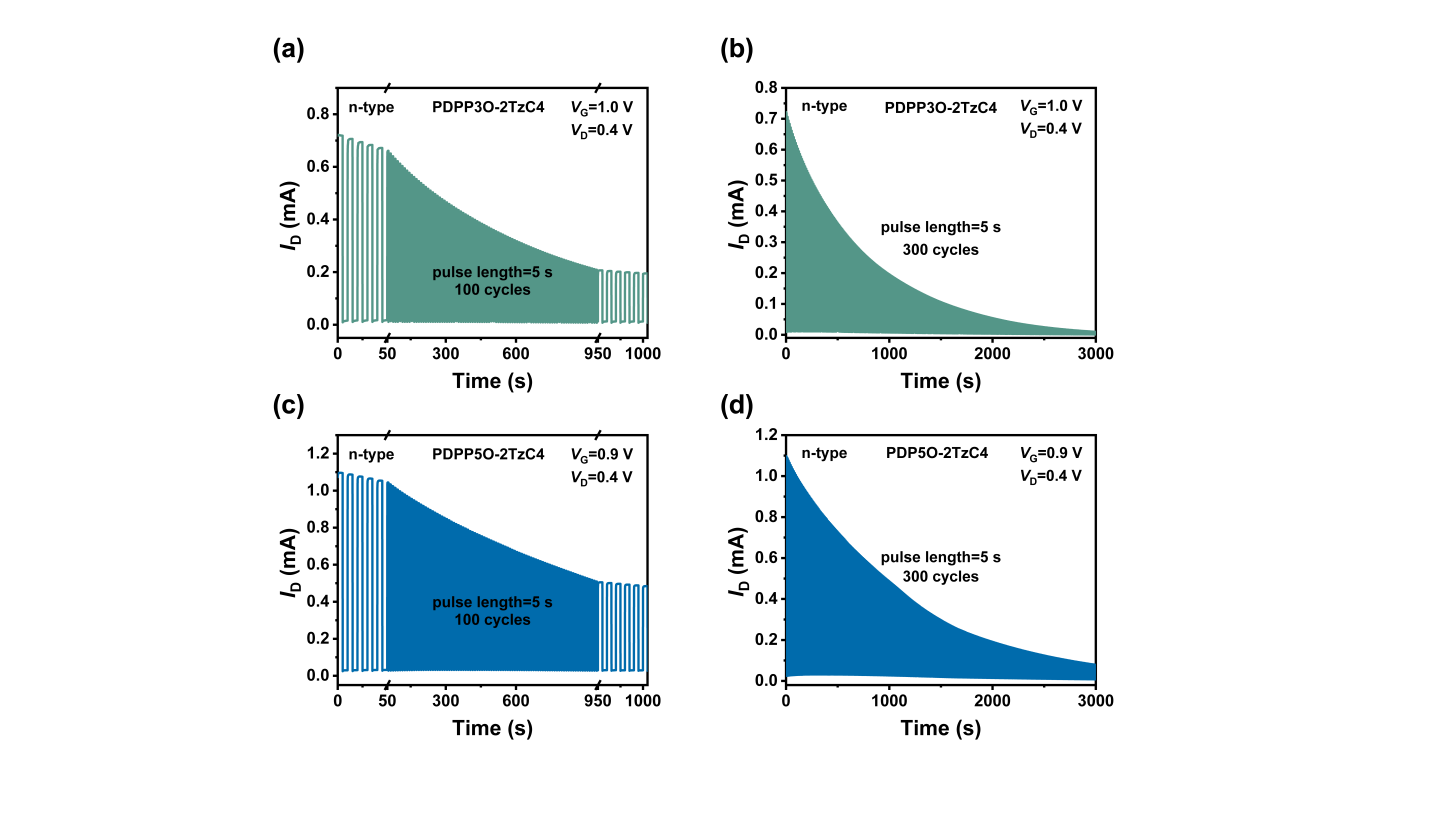


**Figure S19.** **The n-type stability of PDPP3O-2TzC4 and PDPP5O-2TzC4.** Stability of 100 switching cycles for a) PDPP3O-2TzC4 and c) PDPP5O-2TzC4. Tests at 300 switching cycles of b) PDPP3O-2TzC4 and d) PDPP5O-2TzC4.


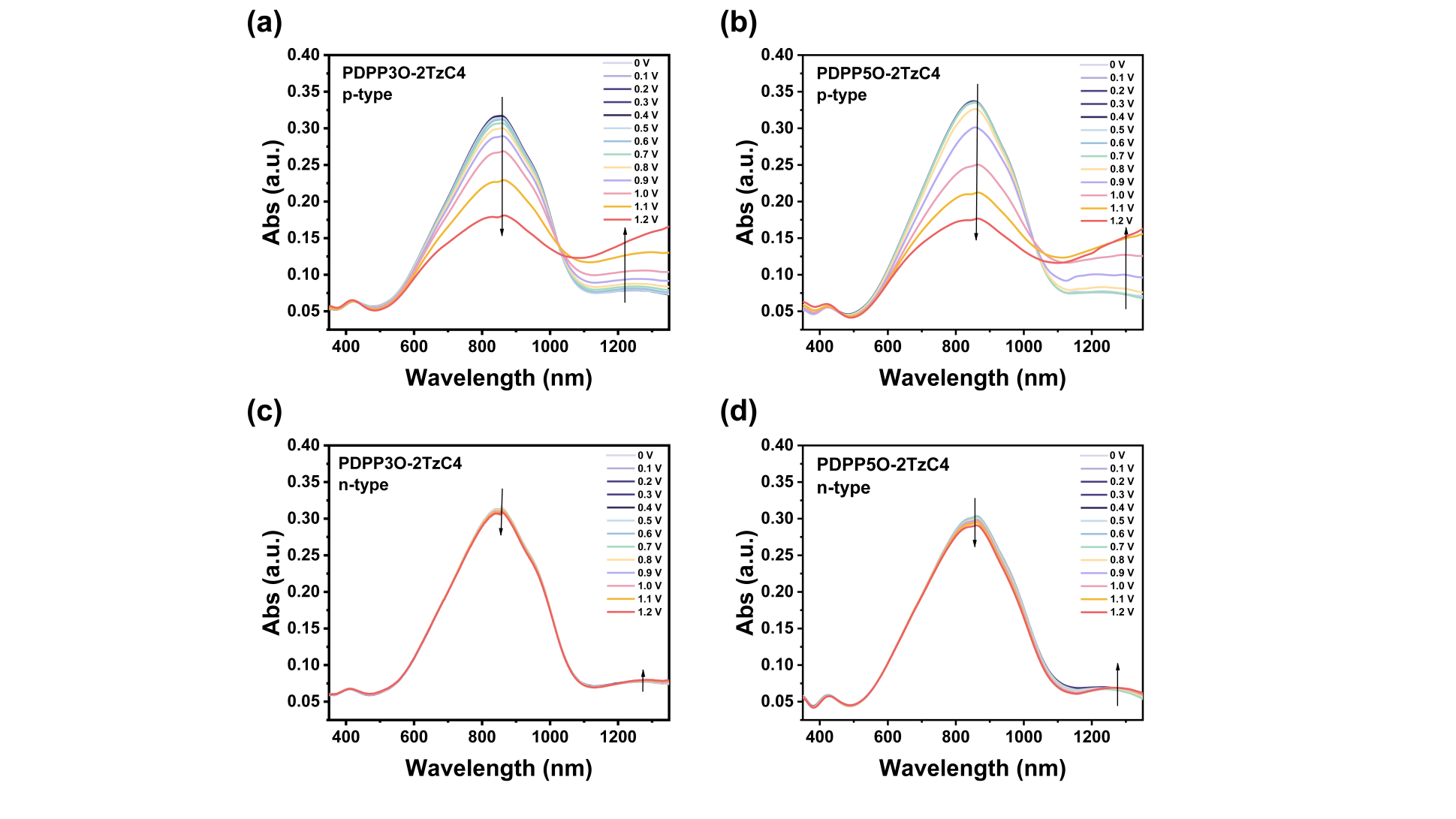


**Figure S20.** Electrochemical spectra of a) p-type and c) n-type PDPP3O-2TzC4 thin films, and b) p-type and d) n-type PDPP5O-2TzC4 thin films.


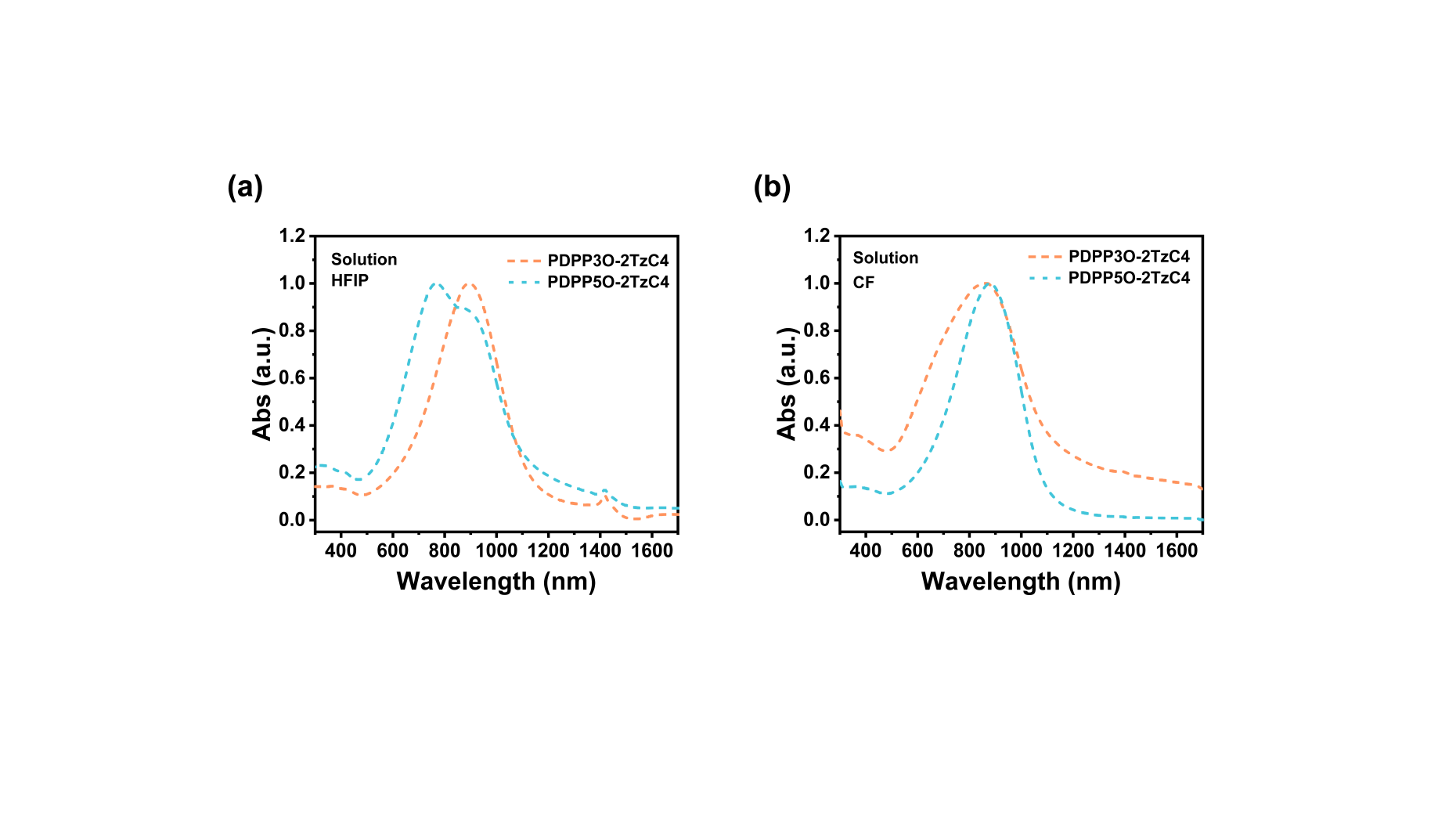


**Figure S21.** UV-vis-NIR absorption spectra of 0.01 mg/mL PDPP3O-2TzC4 and PDPP5O-2TzC4 solutions in a) HFIP and b) chloroform (CF) solvents respectively.


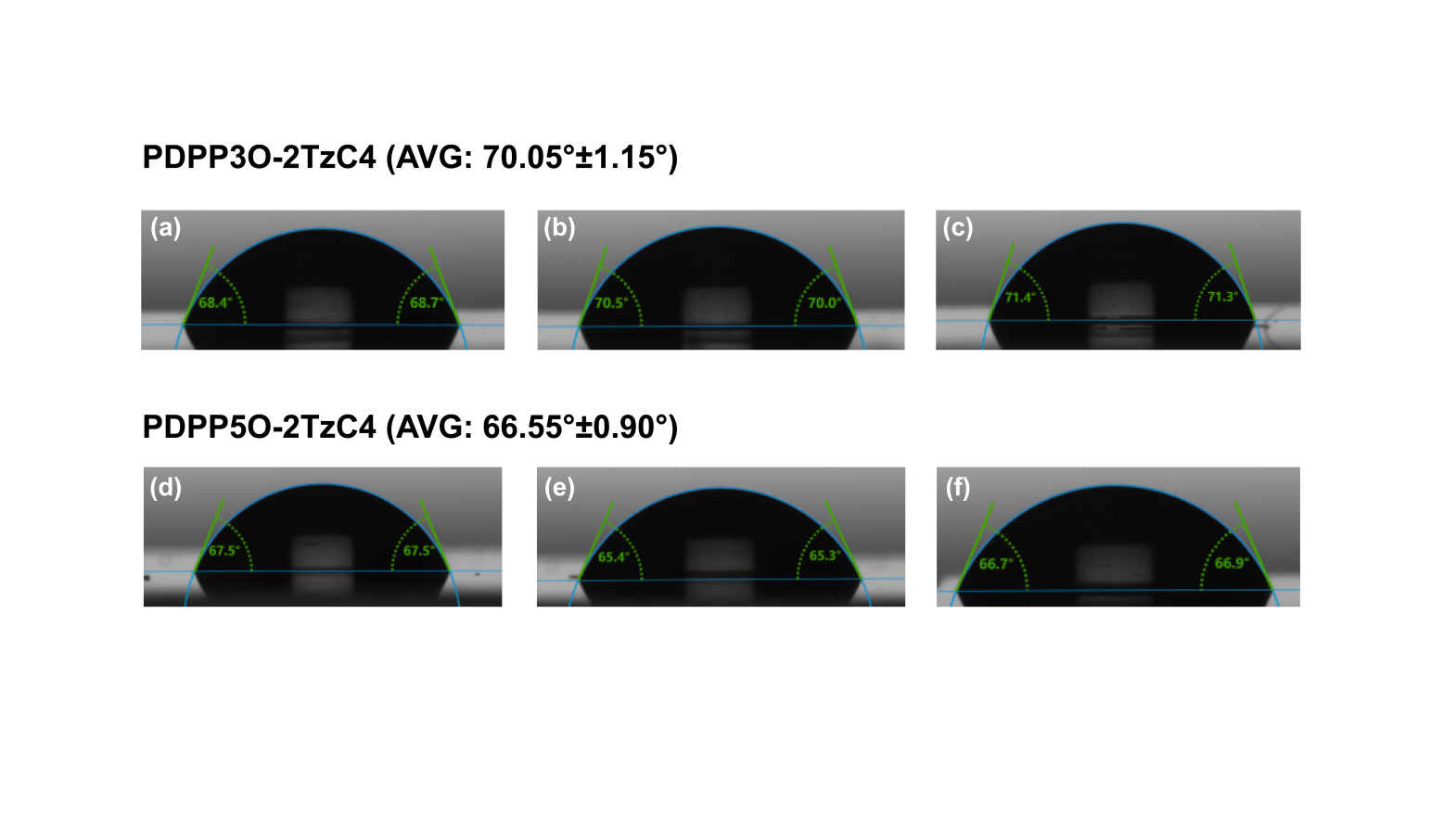


**Figure S22.** a, b, c) Water Contact Angle of PDPP3O-2TzC4 thin films. d, e, f) Water Contact Angle of PDPP5O-2TzC4 thin films.


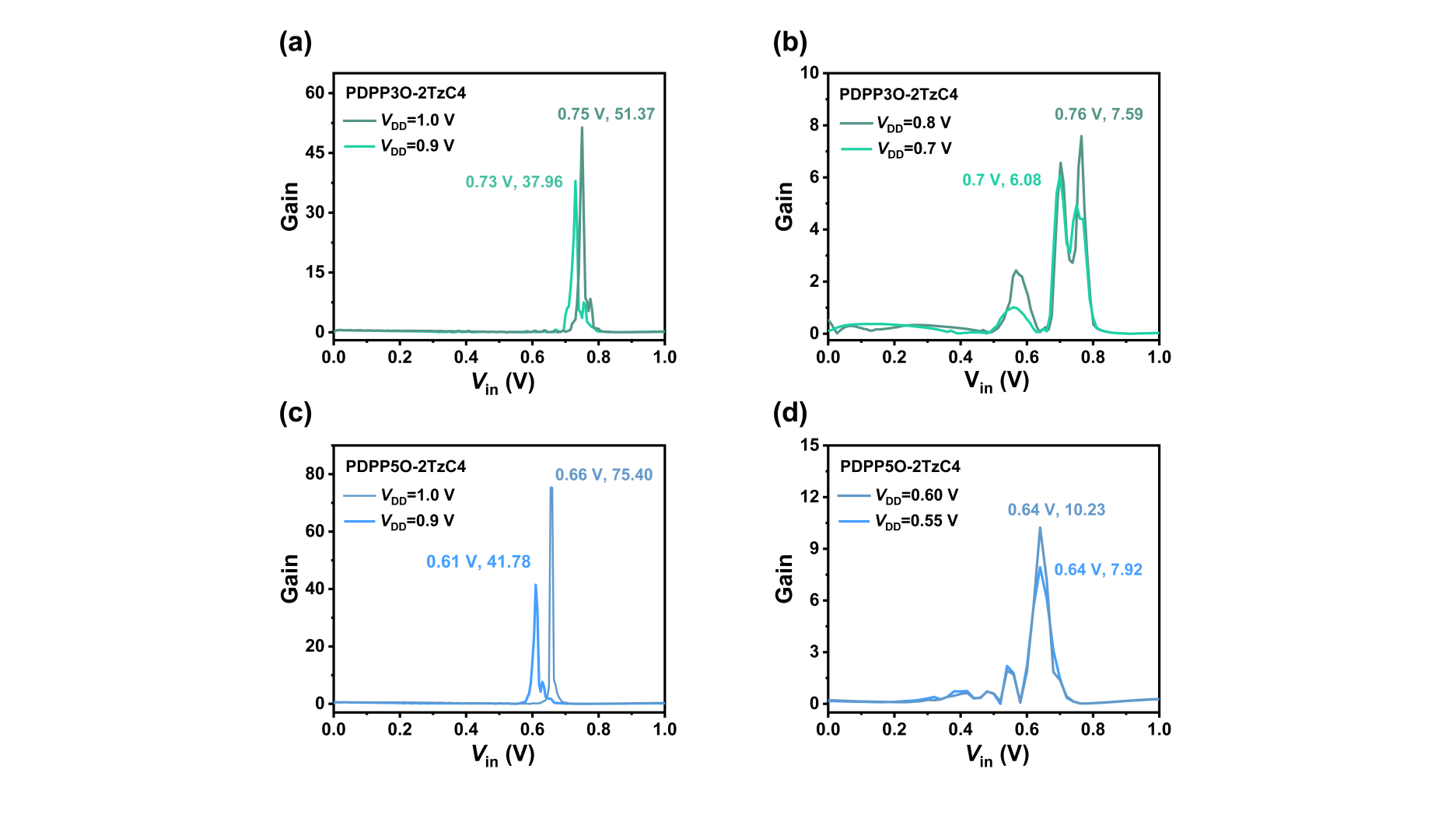


**Figure S23.** Gain curves of single-component inverters based on interdigitated electrodes of PDPP3O-2TzC4 at a) *V*_DD_ = 0.9 V, 1.0 V and b) *V*_DD_ = 0.7 V, 0.8 V, and of PDPP5O-2TzC4 at c) *V*_DD_ = 0.9 V, 1.0 V and d) *V*_DD_ = 0.55 V, 0.60 V.


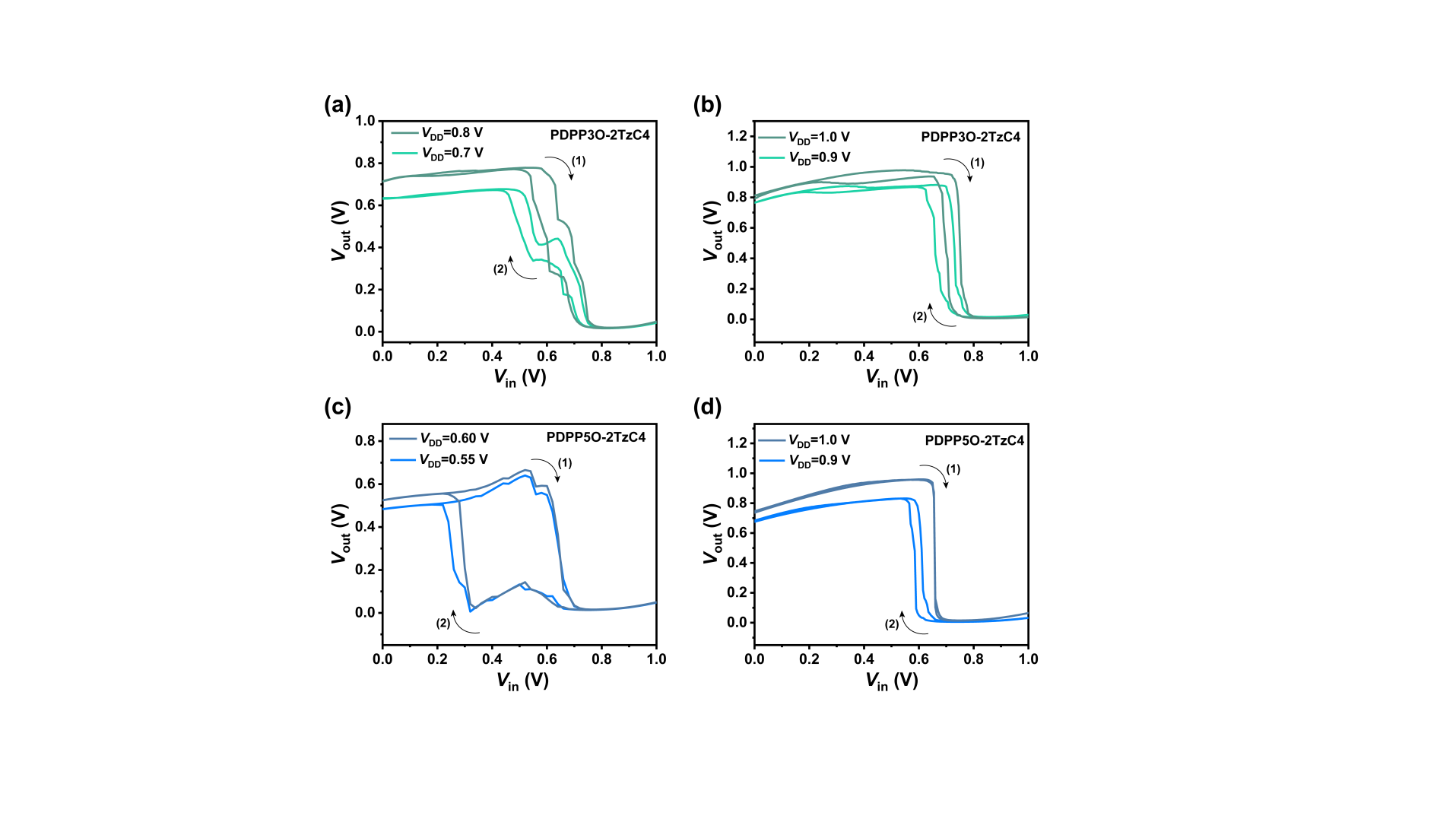


**Figure S24.** Voltage transfer curves of single-component inverters based on interdigitated electrodes of PDPP3O-2TzC4 at a) *V*_DD_ = 0.7 V, 0.8 V and b) *V*_DD_ = 0.9 V, 1.0 V, and of PDPP5O-2TzC4 at c) *V*_DD_ = 0.60 V, 0.55 V and d) *V*_DD_ = 0.9 V, 1.0 V. (1) Forward and (2) Backward voltage sweeps. a) presents supplementary experimental data, where the parameters differ from those in Figure 5c of the main text; b, c, d) show parameters and data that are consistent with the manuscript.


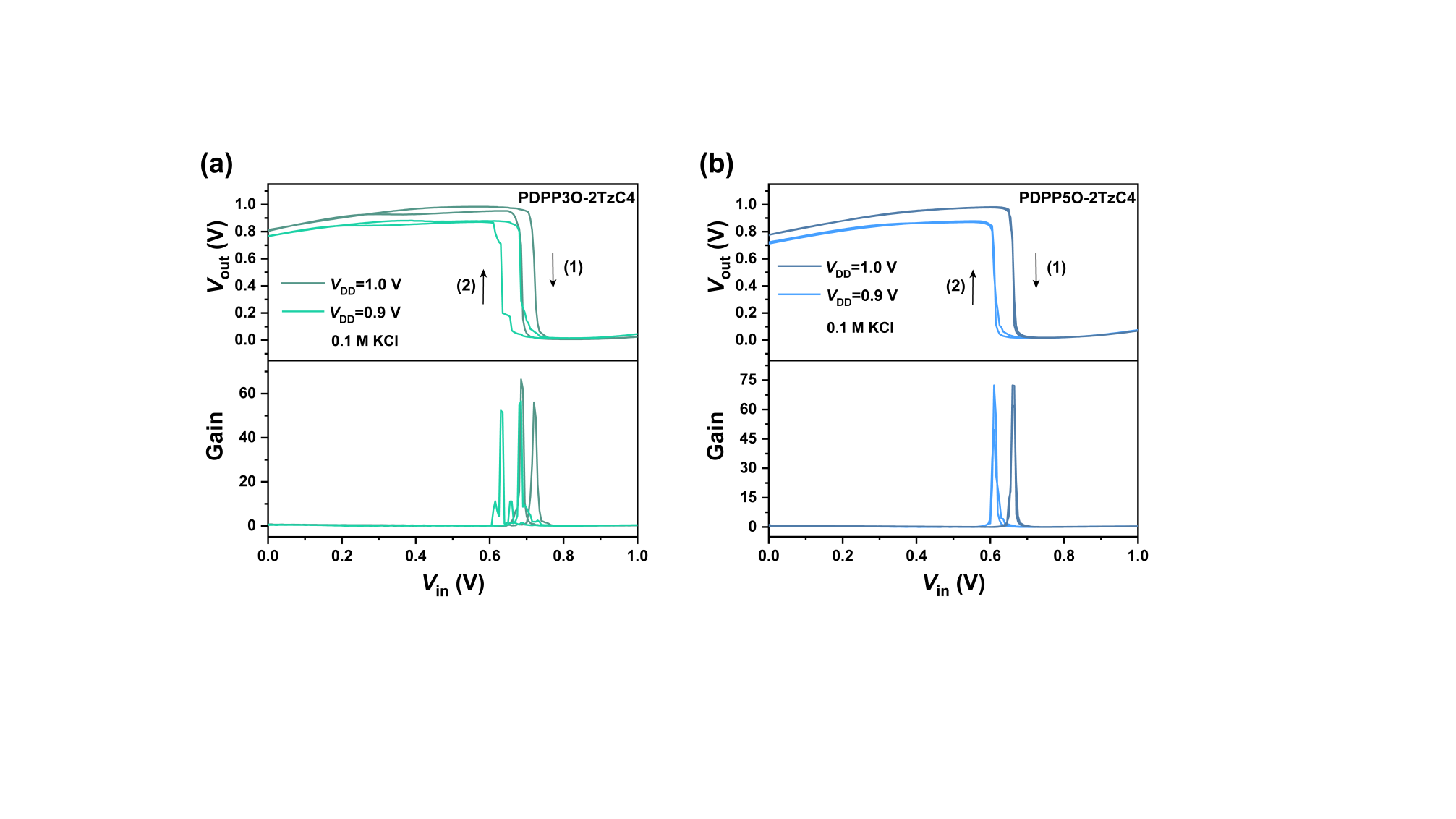


**Figure S25.** Voltage transfer and gain curves of single-component inverters based on a) PDPP3O-2TzC4 and b) PDPP5O-2TzC4, measured in 0.1 M KCl electrolyte. (1) Forward and (2) Backward voltage sweeps. For PDPP3O-2TzC4, the forward-sweep gains are 56.65 V/V at *V*_DD_ = 0.9 V and 56.02 V/V at *V*_DD_ = 1.0 V, while the backward-sweep gains are 52.32 V/V and 66.47 V/V respectively. For PDPP5O-2TzC4, the forward-sweep gains are 72.32 V/V at *V*_DD_ = 0.9 V and 72.33 V/V at *V*_DD_ = 1.0 V, with corresponding backward-sweep gains of 49.61 V/V and 62.10 V/V. Unless otherwise specified, all other devices were characterized using 0.1 M NaCl as the electrolyte.

**
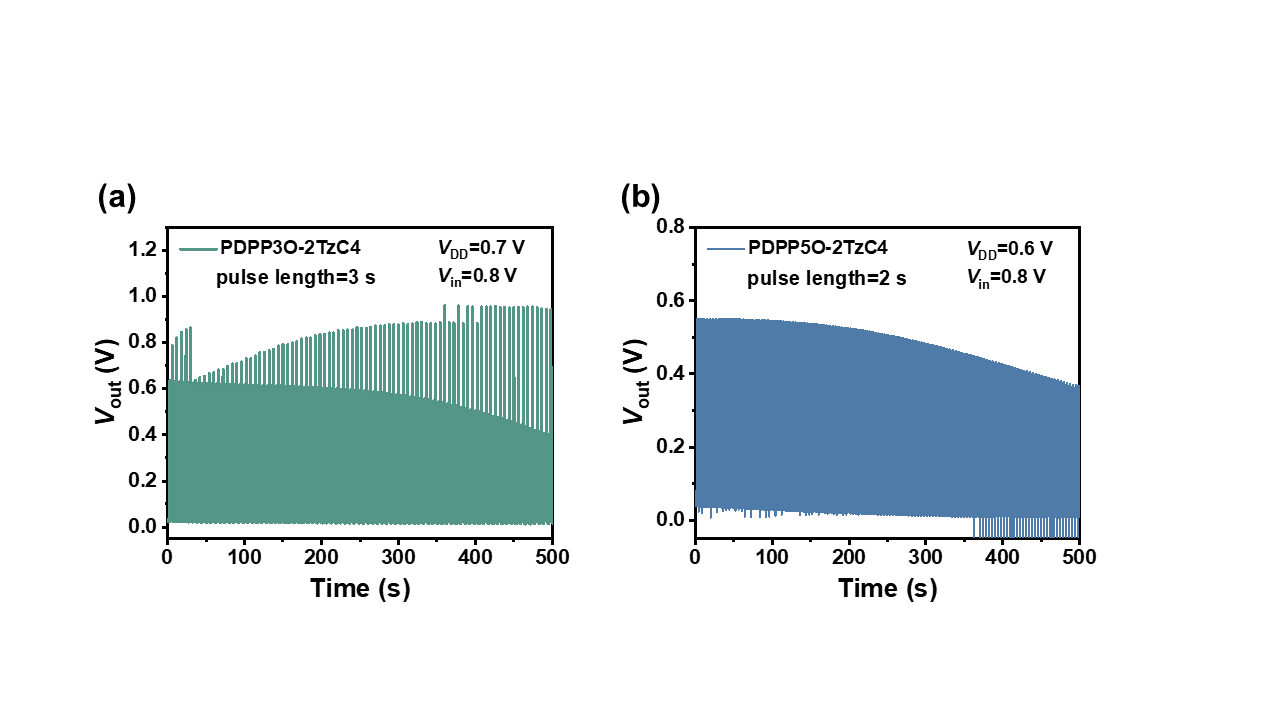
**

**Figure S26.** a) Operational stability of a single-component inverter based on PDPP3O-2TzC4 under a 3 s pulse with *V*_in_ = 0.8 V and a constant *V*_DD_ = 0.7 V. b) Operational stability of a single-component inverter based on PDPP5O-2TzC4 under a 3 s pulse with *V*_in_ = 0.8 V and a constant *V*_DD_ = 0.6 V. The spikes in the plots are likely caused by overshooting during the source meter's voltage switching. They do not reflect the material or device performance.


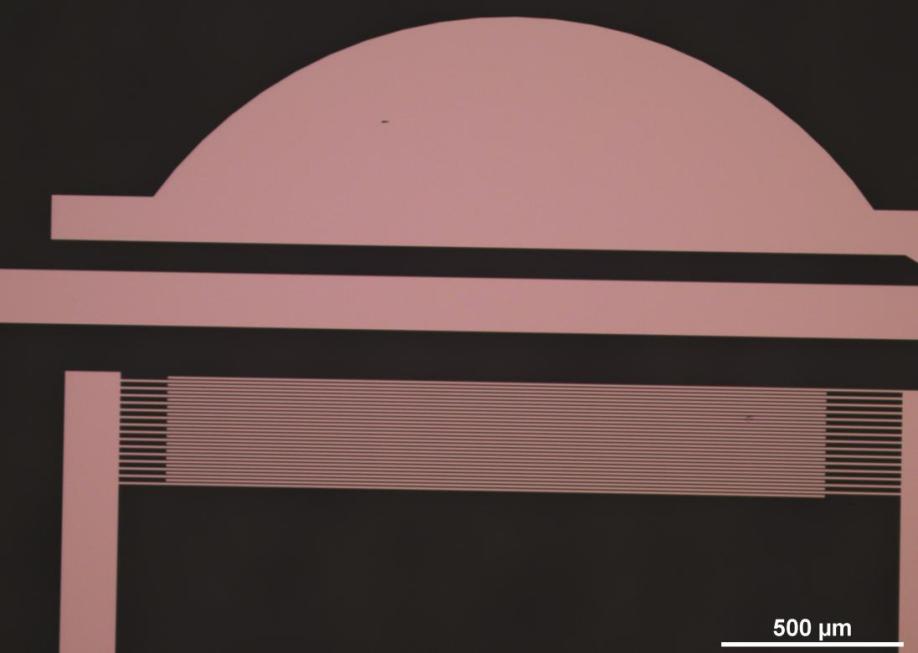


**Figure S27.** Optical image of interdigitated electrodes in metallographic microscopes.

**Table S1.** Performance comparison of several OECT materials.

| **Material** | **type** | ***V*_G_ (V)** | ***I*_on/off_** | ***g*_m,max_ (mS)** | ***g*_m, norm_ (S cm^-1^)** | ***μC** [F V^-1^ cm^-1^ s^-1^]** | ***C** (F cm^-3^)** | **Reference** |
| --- | --- | --- | --- | --- | --- | --- | --- | --- |
| **PDPP3O-2TzC4** | p-type | 0 - -0.8 | > 10^5^ | 28.33 | 0.48 | 1.66 | 134.01 | This work |
|  | n-type | 0 - 1.1 | > 10^5^ | 20.91 | 0.34 | 1.17 | 88.83 |  |
| **PDPP5O-2TzC4** | p-type | 0 - -0.7 | > 10^6^ | 22.68 | 0.42 | 1.27 | 166.67 |  |
|  | n-type | 0 - 1.0 | > 10^6^ | 15.70 | 0.29 | 1.07 | 75.64 |  |
| **p(gNDI-gT2)** | p-type | -0.2 - -0.8 | ~10^2^ | 13.4×10^-3^ | 0.067 | / | / | *Nat. Commun.* **2016**, *7*, 13066 |
|  | n-type | 0 - 0.55 | ~10^3^ | 21.7×10^-3^ | 0.109 | 0.18 | 397 |  |
| **2DPP-OD-TEG** | p-type | 0 - -1.0 | > 10^5^ | 190×10^-6^ | 1.65 | 31.8 | 146.2 (Na^+^) | *Adv. Funct.* Mater. **2021**, *31*, 2102903 |
|  | n-type | 0 - 1.2 | > 10^5^ | 84×10^-6^ | 0.73 | 6.8 | 61.6 (ClO_4_^-^) |  |
| **DHF-gTT** | p-type | 0 - -1.0 | ~ 10^2^ | ~ 0.7 | 2.60 | 11.9 | 41.9 | *Adv. Funct. Mater.* **2024**, 2413112 |
|  | n-type | 0 - 0.7 | ~ 10^3^ | ~ 1.1 | 4.17 | 14.0 | 104.8 |  |
| **DH-gTT** | p-type | 0 - -0.9 | ~ 10^3^ | ~ 0.35 | 0.23 | 5.6 | 71.8 |  |
|  | n-type | 0 - 0.7 | ~ 10^3^ | ~ 0.37 | 1.30 | 5.4 | 134.6 |  |
| **P-6O** | / | 0 - -0.9 | *~* 10^4^ | / | / | / | / | *Adv. Sci.* **2024**, *11*, 2400872 |
|  | n-type | 0 - 0.8 | *>* 10^5^ | 84 | 0.61 | 0.63 | / |  |
| **p(gDPP-V)** | p-type | 0 - -0.8 | ~ 10^7^ | 267.5 | 29 | 204 | 142 | *Sci. Adv.* **2024**, *10*, eadq9405 |
|  | n-type | 0 - 1.0 | ~ 10^7^ | 263.2 | 25 | 102 | 112 |  |
| **2gDPP-RD-V** | p-type | 0 - -0.9 | ~ 10^5^ | 93.2 | 61.3 | 159.6 | 168.3 | *Adv. Mater.* **2025**, 2501041 |
|  | n-type | 0 - (~)1.15 | ~10^4^ | 49.1 | 33.1 | 330.2 | 401.4 |  |
| **PBBTL:BBL(3:1, w:w)** | p-type | 0 - -0.6 | ~ 10^3^ | 0.41 | 0.58 | 2.72 | 124 | *Adv. Mater.* **2022**, *34*, 2206118 |
|  | n-type | 0 - 0.6 | ~ 10^3^ | 0.43 | 0.67 | 1.36 | 168 |  |

**3. References**

[1] F. Weigend, R. Ahlrichs, *Phys. Chem. Chem. Phys.* **2005**, *7*, 3297–3305.

[2] N. Mardirossian, M. Head-Gordon, *The Journal of Chemical Physics* **2016**, *144*, 214110.

[3] M. Bursch, J. Mewes, A. Hansen, S. Grimme, *Angew. Chem.-Int. Edit.* **2022**, *61*, 27.

[4] S. Grimme, C. Bannwarth, P. Shushkov, *J. Chem. Theory Comput.* **2017**, *13*, 1989–2009.

[5] M. Abe, *Chem. Rev.* **2013**, *113*, 7011–7088.

[6] K. Mayer, D. Adams, N. Eedugurala, M. Lockart, P. Mahalingavelar, L. Huang, L. Galuska, E. King, X. Gu, M. Bowman, J. Azoulay, *Cell Rep. Phys. Sci.* **2021**, *2*, 17.

[7] K. Yamaguchi, H. Fukui, T. Fueno, *Chem. Lett.* **1986**, 625–628.

[8] M. Head-Gordon, *Chem. Phys. Lett*. **2003**, *372*, 508–511.
